# Molecularly Imprinted PDMS/MIL@MITpBD Mixed Matrix Membranes for β-Damascone Separation

**DOI:** 10.3390/membranes16100347

**Published:** 2026-10-19

**Authors:** Pei He, Tao Zhang, Xinru Zhang, Shiyun Tang, Jinghui Wang, Yonghong Wang

**Affiliations:** 1 Yunnan Key Laboratory of Tobacco Chemistry, China Tobacco Yunnan Industry Co., Ltd., Kunming 650231, China; zhangtao@ynzy-tobacco.com (T.Z.);; 2 College of Chemistry and Chemical Engineering, Taiyuan University of Technology, Taiyuan 030024, China

**Keywords:** mixed matrix membranes, selective recognition, β-DC separation, dummy template, molecularly imprinted materials

## Abstract

β-damascone (β-DC) has low content in nature and poor thermal stability, so conventional separation methods suffer from low separation efficiency. Pervaporation membrane separation has garnered significant interest owing to its green and energy-saving advantages. However, the design of membranes for efficient β-DC separation remains a challenge. In this study, surface molecularly imprinted material, MIL@MITpBD, was fabricated by in situ growth of a TpBD-OH-ECOEA shell on MIL-101-NH_2_ via keto-enamine condensation, during which (E)-3-(4-chlorobenzoyl)acrylic acid (ECOEA), matching β-DC in steric dimensions, was immobilized in the TpBD-OH shell as a dummy template by hydrogen bonding. After ECOEA removal, MIL@MITpBD with selective imprinted cavities was obtained. The imprinted MIL@MITpBD was then incorporated into polydimethylsiloxane (PDMS) polymer matrix to fabricate mixed matrix membranes (MMMs) for β-DC separation. Separation performance evaluation revealed a total flux (*J*) of 587 g m^−2^ h^−1^ and a separation factor (*β*_β-DC/H2O_) of 353.2 at 50 °C with a feed concentration of 100 ppm, which was attributed to the synergistic effect of the selective recognition of imprinted MIL@MITpBD and abundant hydrogen-bonding sites. Furthermore, during a 168 h continuous operation test, the membrane maintained 593.8 g m^−2^ h^−1^ *J* and 341 *β*_β-DC/H2O_, evidencing good long-term operational stability. This study provides an effective strategy for the design of pervaporation membranes for trace organic compounds.

## 1. Introduction

β-damascone (β-DC) possesses an extremely low odor threshold, allowing it to impart a pronounced rosy and fruity sweetness to products even at trace concentrations [1]. Natural β-DC, as a typical “high–impact” aroma chemical, is only used in high–end fragrance and flavor markets but is recognized as a key odor-contributing component in the fields of cosmetics, food, and daily necessities. However, β-DC has a low natural abundance in plants and relatively poor thermal stability, making it highly susceptible to degradation or structural isomerization during extraction and separation processes, thereby leading to a decline in product quality. To date, there are few reports on the separation of β-DC from plants. Distillation and solvent extraction are used as the main separation methods for active ingredients in natural products. Distillation suffers from high energy consumption and is prone to damaging heat-sensitive components [2], while solvent extraction faces issues of solvent residue and high energy consumption, making it difficult to balance both environmental friendliness and economic efficiency [3]. More importantly, both methods are unfavorable for the separation of heat-sensitive substances such as β-DC. In contrast, pervaporation membrane separation demonstrates significant potential for the effective separation of aromatic active components due to its mild process conditions, lack of requirement for additional reagents, and low energy consumption [4]. Pervaporation membranes can be mainly classified into polymeric membranes [5], inorganic membranes [6,7], and mixed matrix membranes (MMMs) [8,9] according to the material type. MMMs combine the advantages of polymeric and inorganic membranes, breaking the trade-off limitation of polymeric membranes, and have therefore attracted widespread attention in separating organic compounds by pervaporation. However, conventional membrane materials still struggle to achieve efficient recognition and separation of specific trace components. Fabricating membranes with high selective recognition capability is key for the high–efficiency separation of β-DC by pervaporation.

Molecular imprinting technology (MIT) enables the precise separation of target molecules by constructing three–dimensional cavities with selective recognition sites [10,11,12]. Integrating MIT with membrane separation to fabricate molecularly imprinted membranes (MIMs) can combine high selectivity with high flux, thereby achieving a directed, stable, and economical separation process in complex systems [13]. In recent years, significant progress has been made in research on MIMs. Chai et al. [14] fabricated PVDF@Ag@P@MIMs by constructing a hydrophilic crosslinking layer on the surface of polyvinylidene fluoride (PVDF) using tannic acid and polyethyleneimine, followed by the formation of an imprinted layer on the surface via dopamine self-polymerization with bisphenol A (BPA) as the template molecule, which exhibited an optimal adsorption of 33.41 mg·g^−1^ for BPA. Yan et al. [13] fabricated ribavirin dual-imprinted MOFs membranes (R-DIIMMs) by introducing ribavirin molecules as a template into UiO-66-derived MOFs materials containing −NH_2_ and −OH groups via an in situ synthesis strategy, followed by a two-step imprinting process involving rotary evaporation and a sol–gel technique. The resulting membranes were employed for ribavirin separation, exhibiting a high adsorption capacity (46.46 mg·g^−1^) and excellent selectivity (more than 6) toward ribavirin. Gao et al. [15] fabricated a dual-imprinted membrane with synergistic covalent and non-covalent interactions by using luteolin (LTL) as a template on a chitosan (CS) membrane substrate. The first imprinting sites were formed via dopamine self-polymerization, followed by the introduction of bifunctional monomers via atom transfer radical polymerization (ATRP) to construct the second imprinting sites. The resulting membrane exhibited an adsorption capacity of 49.63 mg·g^−1^ for LTL and a selectivity (*α*_LTL/AGN_) of 6.20. These studies demonstrate that incorporating inorganic or hybrid materials with imprinted structures in membranes is an effective approach for constructing high-performance molecularly imprinted mixed matrix membranes, exhibiting great potential for the selective separation of trace organic compounds. However, for β-DC, which possesses few reactive functional groups and poor thermal stability, designing an appropriate imprinting strategy to construct highly selective recognition sites remains a critical challenge that urgently needs to be addressed.

Dummy-template molecularly imprinted membranes may provide an effective approach to remedy the shortage of available imprinting sites for β-DC recognition. The dummy–template strategy refers to the use of a compound that is structurally similar to the target molecule but contains more reactive functional groups or is more available, rather than the target molecule itself, as a template molecule to construct imprinted cavities [16]. The introduction of dummy templates into the fabrication of molecularly imprinted membranes can endow the membranes with selective recognition ability toward target molecules while maintaining high flux. In recent years, significant progress has been made in the field of selective separation using dummy-template molecular imprinting technology. Zhou et al. [16] used teicoplanin (TEC) as a dummy template to prepare a dummy-template molecularly imprinted polymer (SMIP) for vancomycin (VCM) and norvancomycin (NVCM). The VCM recovery of the SMIP was 101.4%, which was significantly higher than that on the non-imprinted polymer (NIP) (69.1%), demonstrating the selective recognition ability of the imprinted cavities. Wang et al. [17] fabricated a dihydroxybiphenyl molecularly imprinted polymer (DHBP-MIP) using non-toxic 4,4’-dihydroxybiphenyl (DHBP) as a dummy template to substitute for highly toxic polychlorinated biphenyls (PCBs). The resulting imprinted polymer exhibited excellent recognition ability toward PCBs, with an adsorption capacity of 8.43 mg·g^−1^. 

In this work, (E)-3-(4-chlorobenzoyl)acrylic acid (ECOEA), which possesses abundant reactive functional groups and exhibits a size and shape similar to β-DC, is selected as the dummy template to fabricate the imprinted material. The chemical structure comparison between ECOEA and β-DC is displayed in Figure S1 of the Supporting Information, and this strategy creates imprinted cavities that are geometrically complementary to β-DC in shape and size. First, the shell-structured MITpBD-ECOEA was synthesized on the surface layer of MIL-101-NH_2_, where the ECOEA was immobilized in the shell (MITpBD-ECOEA). Subsequently, the ECOEA was removed to obtain the MIL@MITpBD imprinted material. Finally, the core–shell MIL@MITpBD was doped into polydimethylsiloxane (PDMS) to prepare PDMS/MIL@MITpBD mixed matrix membranes, which were then used for the β-DC separation. The effects of the preparation conditions and operating conditions of PDMS/MIL@MITpBD on pervaporation performance for β-DC were systematically investigated. The stability of PDMS/MIL@MITpBD was evaluated via a 168 h continuous operation test, and the separation mechanism was elucidated.

## 2. Experimental Methods

### 2.1. Materials

The details on the chemicals and materials used are provided in Section S2.1 of the Supporting Information. 

### 2.2. Synthesis of MIL@MITpBD

Firstly, 32.4 mg of 3,3’-dihydroxybenzidine (BD-OH) and 20 mg of (E)-3-(4-chlorobenzoyl)acrylic acid (ECOEA) as a dummy template of the target molecule (β-damascone, β-DC) were added to 4 mL of a mixed solvent of 1,3,5-trimethylbenzene and 1,4-dioxane (*v*/*v*, 1:1) and then stirred for 30 min to form BD-OH-ECOEA by hydrogen bonding. Subsequently, 20 mg of MIL-101-NH_2_ and 21 mg of 1,3,5-triformylphloroglucinol (Tp) were introduced into the above solution. The mix solution was then transferred to a Schlenk tube and ultrasonicated for 10 min, after which 0.4 mL of aqueous HAc (6 mol L^−1^) was introduced as a catalyst. The tube was evacuated by three freeze–pump–thaw cycles and heated at 120 °C for 72 h, during which Tp and BD-OH-ECOEA condensed on the MIL-101-NH_2_ surface to grow the TpBD-OH-ECOEA shell while anchoring the template. After cooling, the template-containing intermediate MIL@TpBD-ECOEA was obtained. The ECOEA was then removed by washing with a MeOH/HAc mixed solvent (*v*/*v*, 9:1) until the absorption peaks of the eluate, monitored by UV-vis spectrophotometry, no longer changed appreciably, indicating the removal of ECOEA. After washing with methanol and vacuum drying at 80 °C, MIL@MITpBD was obtained. To tune the thickness of the imprinting layer, the amounts of Tp (5.25, 10.5, 21, and 42 mg) and BD-OH (8.1, 16.2, 32.4, and 64.8 mg) were increased proportionally, and the resulting samples were designated as MIL@MITpBD(X) (X = 1, 2, 3, and 4). Additionally, the syntheses of MIL-101-NH_2_, TpBD-OH, and the non-imprinted counterpart MIL@NITpBD are described in Sections S2.2, S2.3, and S2.4 of the Supporting Information, respectively.

### 2.3. Preparation of PDMS/MIL@MITpBD

**Scheme 1:**
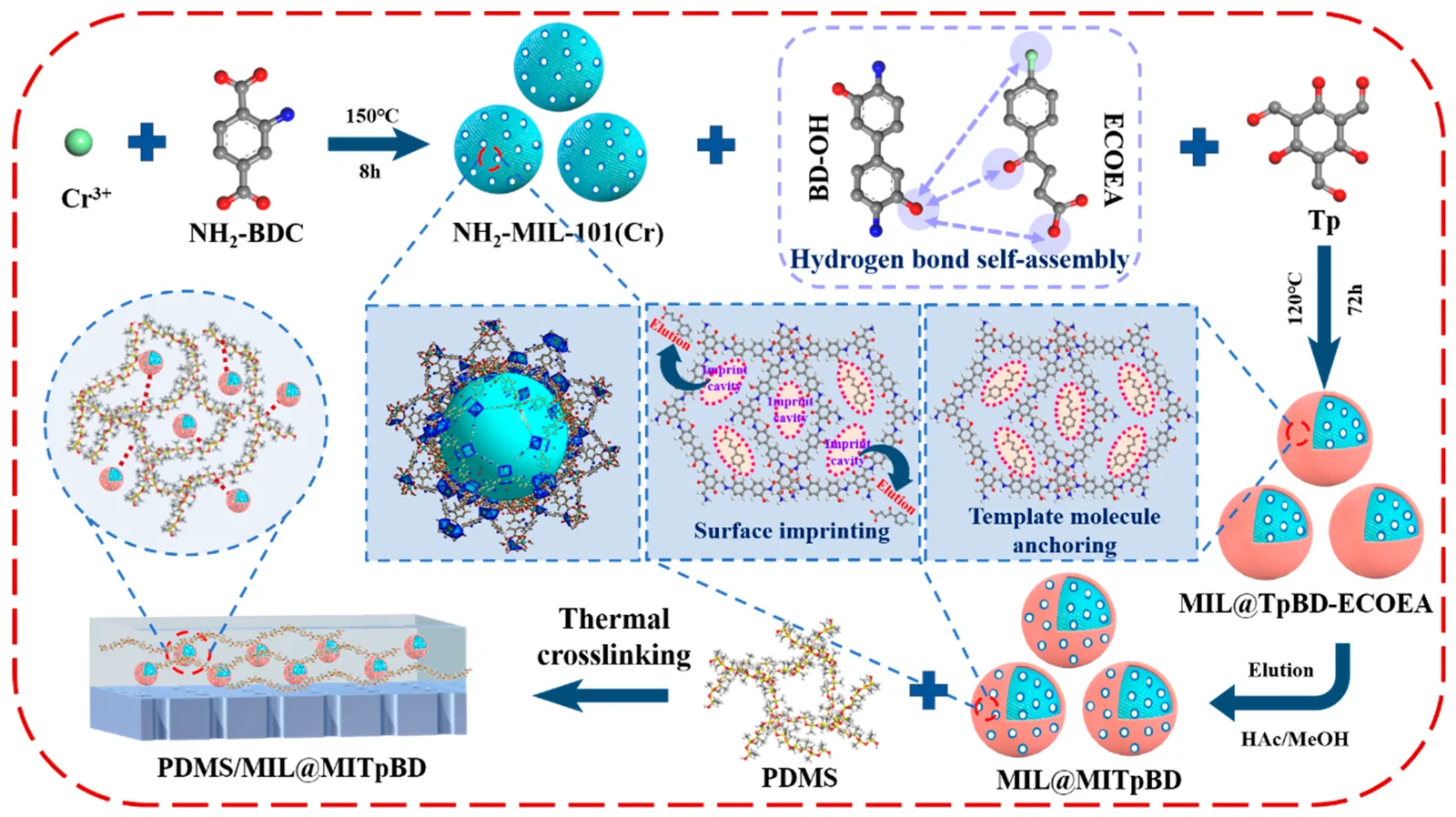
Fabrication schematic of PDMS/MIL@MITpBD.

The fabrication of PDMS/MIL@MITpBD-Y MMMs (where Y represents the weight fraction of MIL@MITpBD relative to total PDMS) is illustrated in Scheme 1. First, 0.2 g of polydimethylsiloxane (PDMS) and 1.75 g of *n*-hexane were mixed and stirred for 2 h to fully dissolve the PDMS. Then, a certain amount of MIL@MITpBD (loading of 1, 3, 5, and 7 wt% relative to the total PDMS mass) was added into the PDMS solution and stirred for 2 h to facilitate dispersion. Subsequently, another 0.8 g of PDMS was added and stirred, ensuring uniform blending of MIL@MITpBD with PDMS. Subsequently, tetraethyl orthosilicate (TEOS) and dibutyltin dilaurate (DBTDL) were added in respective amounts of 0.1666 g and 0.0166 g for pre-crosslinking at room temperature. The viscosity of the casting solution was monitored, and when it reached a suitable level, the solution was poured onto a polysulfone (PSF) support layer controlling the wet thickness at 200 μm by a casting knife. After casting, the membrane was left at room temperature for 8 h and then thermally crosslinked (12 h, 24 h, 32 h and 48 h) in a vacuum oven to afford the PDMS/MIL@MITpBD-Y MMMs (Y = 1, 3, 5, and 7). When investigating the influence of membrane thickness on β-DC separation, the wet thickness of membranes was controlled at 50, 100, 150, 200, and 250 μm, respectively.

### 2.4. Pervaporation Experiment and Related Formulas

A custom-built pervaporation setup was employed; its details are described in Section S2.6 of the Supporting Information.

### 2.5. Characterization

Details on the structural characterization (Section S2.7) and solvent uptake tests (Section S2.8) are available in the Supporting Information.

## 3. Results and Discussion

### 3.1. Characterization of MIL@MITpBD

MIL@MITpBD was fabricated via a core–shell strategy combined with surface molecular imprinting, as shown in Scheme 1. The TpBD-OH-ECOEA was grown in situ via keto-enamine condensation onto the surface of an amino-functionalized MOF, MIL-101-NH_2_, which served as the core, to form the imprinting shell. During this growth, ECOEA, which is spatially and size-matched to β-DC, was employed as a dummy template and anchored into the growing shell through the interaction of hydrogen bonding between ECOEA and BD-OH. ECOEA was subsequently eluted from TpBD-OH-ECOEA, and MIL@MITpBD with imprinting cavities complementary to β-DC was thereby synthesized. The chemical structures of β-DC and ECOEA are given in Figure S1. 

**Figure 1:**
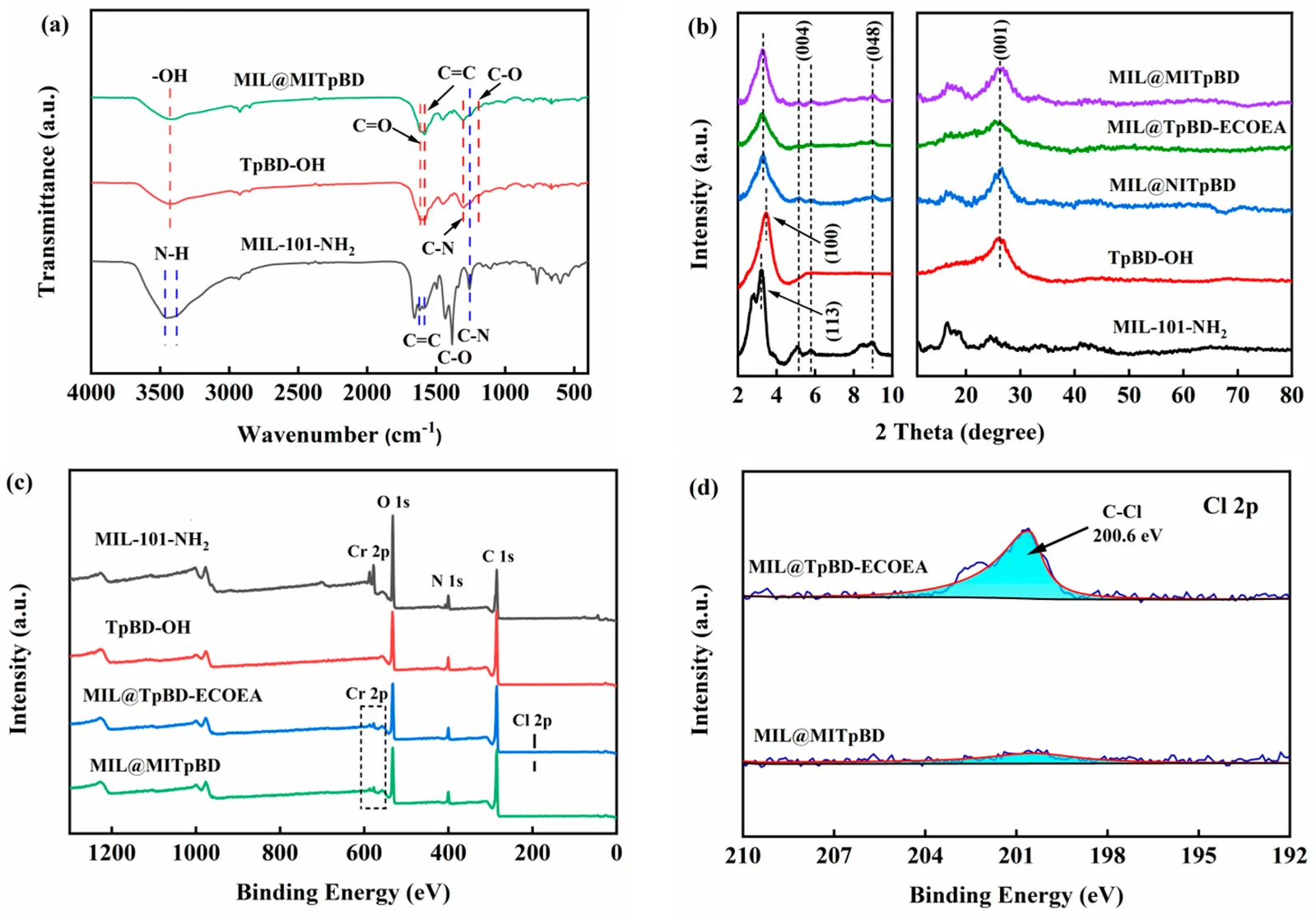
(**a**) FTIR of MIL-101-NH_2_, TpBD-OH and MIL@MITpBD; (**b**) XRD patterns of MIL-101-NH_2_, TpBD-OH, MIL@NITpBD, MIL@TpBD-ECOEA and MIL@MITpBD; (**c**) overall XPS survey spectrum of MIL-101-NH_2_, TpBD-OH, MIL@TpBD-ECOEA and MIL@MITpBD; (**d**) high-resolution XPS spectra of Cl 2p.

The FTIR spectrum of the MIL@MITpBD core–shell material is shown in Figure 1a. The characteristic peaks of TpBD-OH (C=C at 1584 cm^−1^ and C=O at 1612 cm^−1^) and of MIL-101-NH_2_ (C–N at 1257 cm^−1^) appear simultaneously, indicating that the TpBD-OH shell has been successfully coated on the surface of MIL-101-NH_2_ to form a core–shell structure [18,19]. As shown in Figure S3, the FTIR spectrum of ECOEA shows a characteristic C–Cl absorption peak at 760 cm^−1^. After ECOEA was immobilized through hydrogen bonding, this peak shifted to 745 cm^−1^ for MIL@TpBD-ECOEA. Upon template elution, the C–Cl peak disappeared for MIL@MITpBD, indicating successful removal of ECOEA. The XRD pattern of MIL@MITpBD in Figure 1b exhibits the diffraction peaks corresponding to the (113), (004), and (048) planes of MIL-101-NH_2_ together with the (100) and (001) planes of TpBD-OH, indicating the successful formation of the MIL@MITpBD core–shell structure. The decreased peak intensity of MIL@TpBD-ECOEA at 3.26° and 25.80° is attributed to the steric hindrance of ECOEA, which disrupts the ordered π-π stacking of TpBD-OH and thereby reduces the crystallinity, further confirming the successful incorporation of ECOEA. After template elution, the intensity of the MIL@MITpBD peaks at 3.26° and 26.57° was enhanced owing to π-π conjugation. As depicted in Figure 1c,d, the XPS spectrum of MIL@MITpBD exhibits the Cr 2p signal characteristic of MIL-101-NH_2_. The high-resolution Cl 2p spectrum of MIL@TpBD-ECOEA shows a Cl peak at 200.6 eV, indicating that ECOEA was successfully anchored within TpBD-OH through hydrogen bonding. In contrast, the intensity of the Cl peak of MIL@MITpBD was significantly reduced, indicating that the ECOEA molecules had been successfully eluted. 

**Figure 2:**
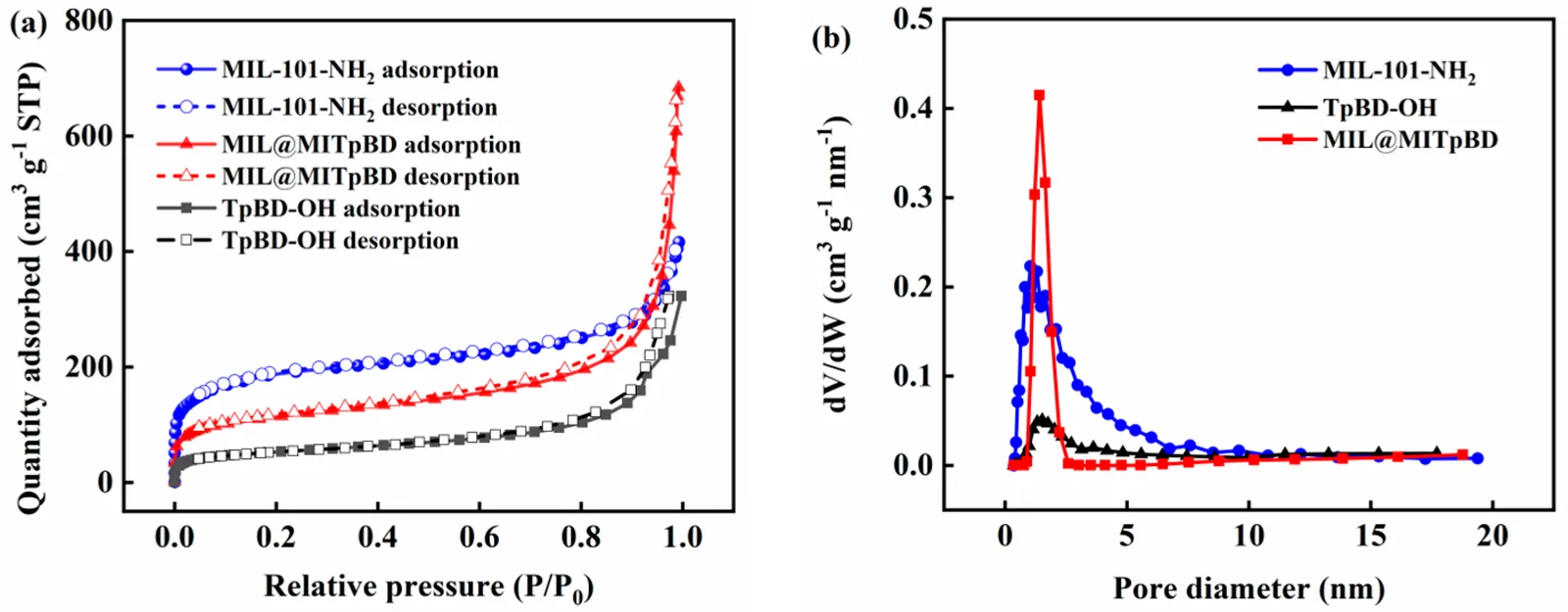

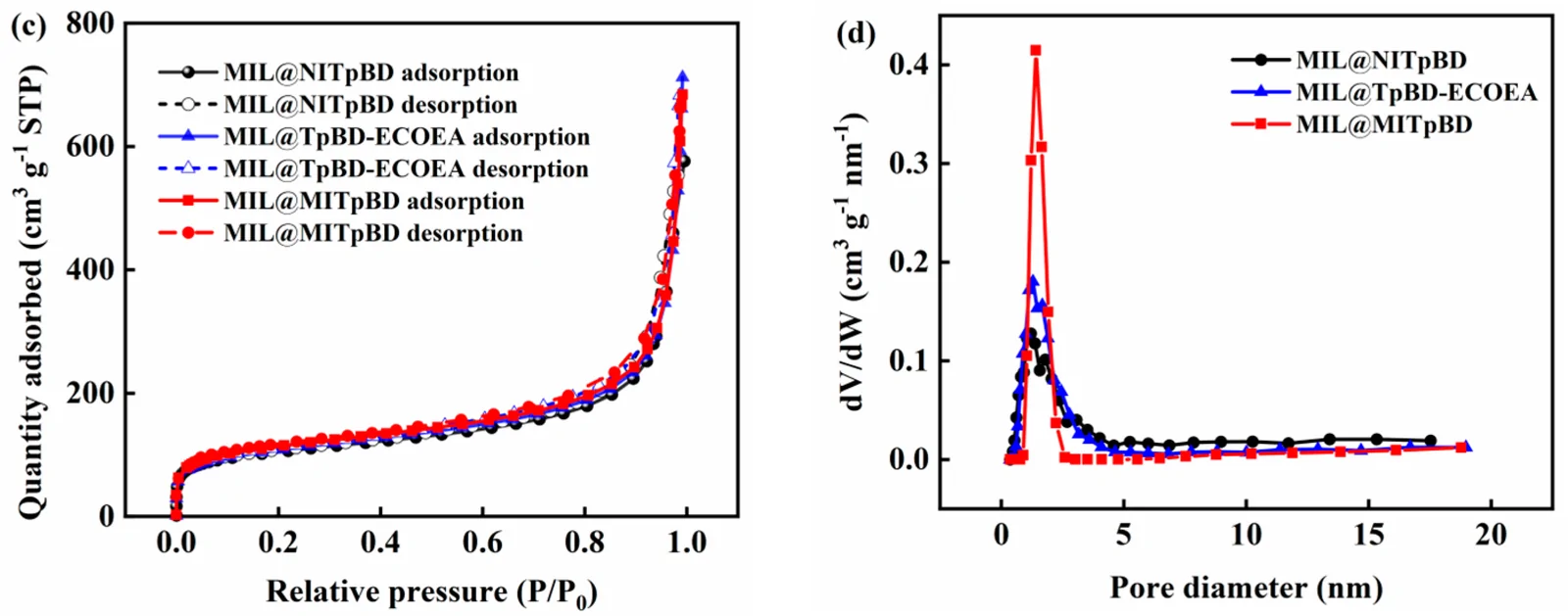
(**a**) N_2_ adsorption and desorption curves and (**b**) pore size distribution of MIL-101-NH_2_, TpBD-OH and MIL@MITpBD; (**c**) N_2_ adsorption and desorption curves and (**d**) pore size distribution of MIL@NITpBD, MIL@TpBD-ECOEA and MIL@MITpBD.

**Figure 3:**
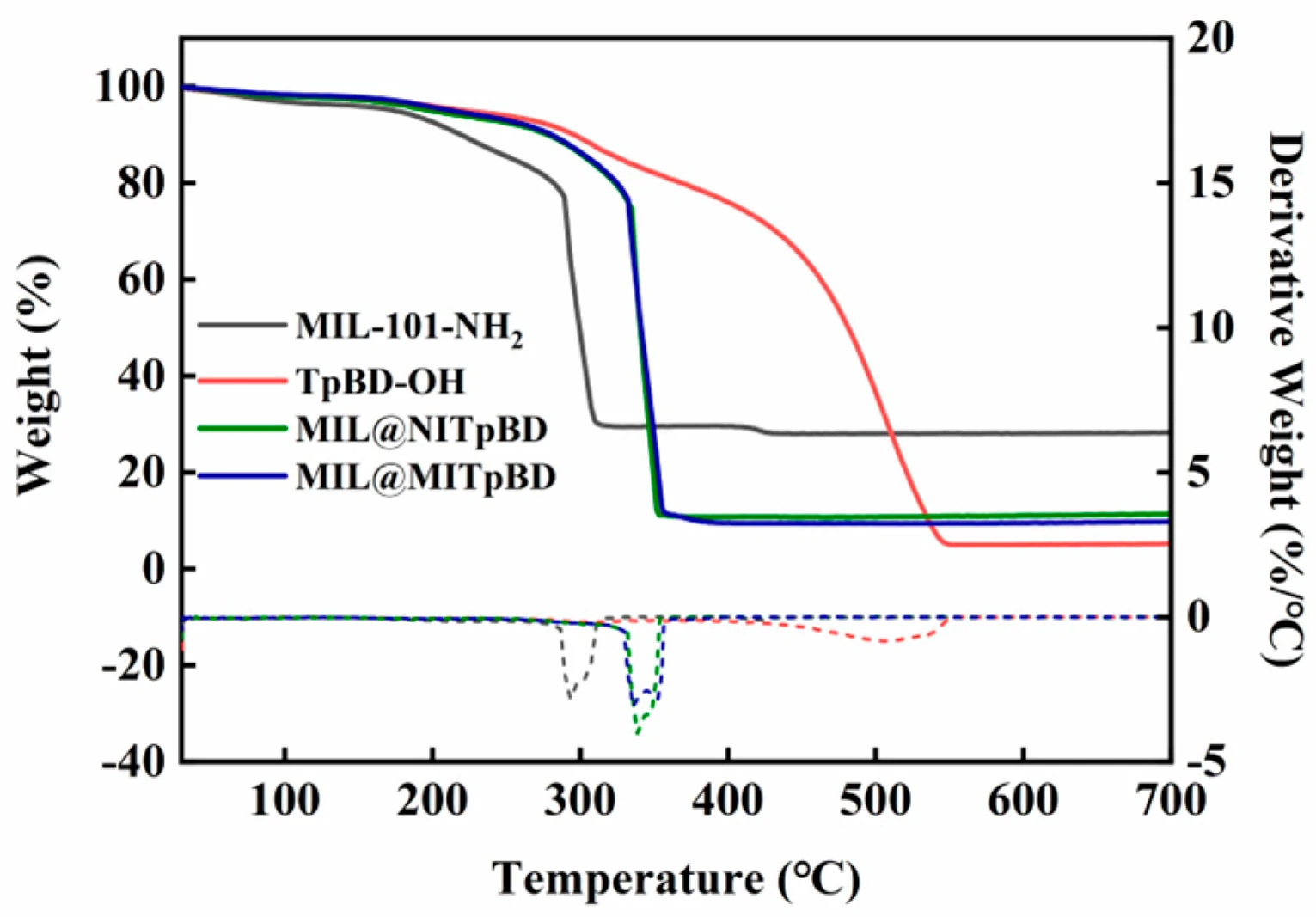
TGA/DTG curves of MIL-101-NH_2_, TpBD-OH, MIL@NITpBD and MIL@MITpBD.

As shown in Figure S4, the stacking structure of MIL@TpBD-ECOEA became relatively loose owing to the steric hindrance of the template molecule ECOEA. After ECOEA removal and the formation of imprinting cavities, MIL@MITpBD well retained its three-dimensional porous sponge-like structure, and the interwoven nanorod network became more open and continuous, indicating that template removal did not destroy the material structure. Furthermore, the BET surface areas and pore size distributions for MIL-101-NH_2_, TpBD-OH, and MIL@MITpBD are summarized in Figure 2a,b and Table S1. All three materials display Type IV adsorption isotherms, indicating the characteristic of containing both micropores and mesopores. As shown in Table S1, MIL@MITpBD exhibits a BET surface area of 405.88 m^2^ g^−1^ and an average pore diameter of 10.42 nm, integrating the porosity characteristics of both MIL-101-NH_2_ and TpBD-OH. Notably, the total pore volume of MIL@MITpBD is larger than those of MIL-101-NH_2_ and TpBD-OH, which can be attributed to the generation of new interstitial and interconnected pores during the growth of TpBD-OH on the surface of MIL-101-NH_2_. Moreover, Figure 2c,d compare the changes in BET surface area and pore structure before and after imprinting. Compared with the template-containing MIL@TpBD-ECOEA, the template-free MIL@MITpBD exhibits a larger specific surface area but a reduced average pore diameter, which is because the removal of the template molecules left imprinting cavities. The diameter of the imprinting cavities of MIL@MITpBD is about 1.4 nm, close to the kinetic diameter of the dummy template ECOEA (1.2 nm). Meanwhile, the average pore diameter decreased owing to the generation of imprinting micropores. The temperature denoting the maximum weight-loss rate (336 °C) of the core–shell MIL@MITpBD lies between those of MIL-101-NH_2_ and TpBD-OH in Figure 3, reflecting a synergistic effect resulting from the combination of MIL-101-NH_2_ and TpBD-OH. Moreover, the thermal stability of the core–shell material is significantly higher than that of the MIL-101-NH_2_ core, because the TpBD-OH shell provides physical protection to the core and retards the collapse of the MIL-101-NH_2_ framework, thereby further confirming the successful coating of the COF shell onto the MOF surface.

### 3.2. Characterization of PDMS/MIL@MITpBD

**Figure 4:**
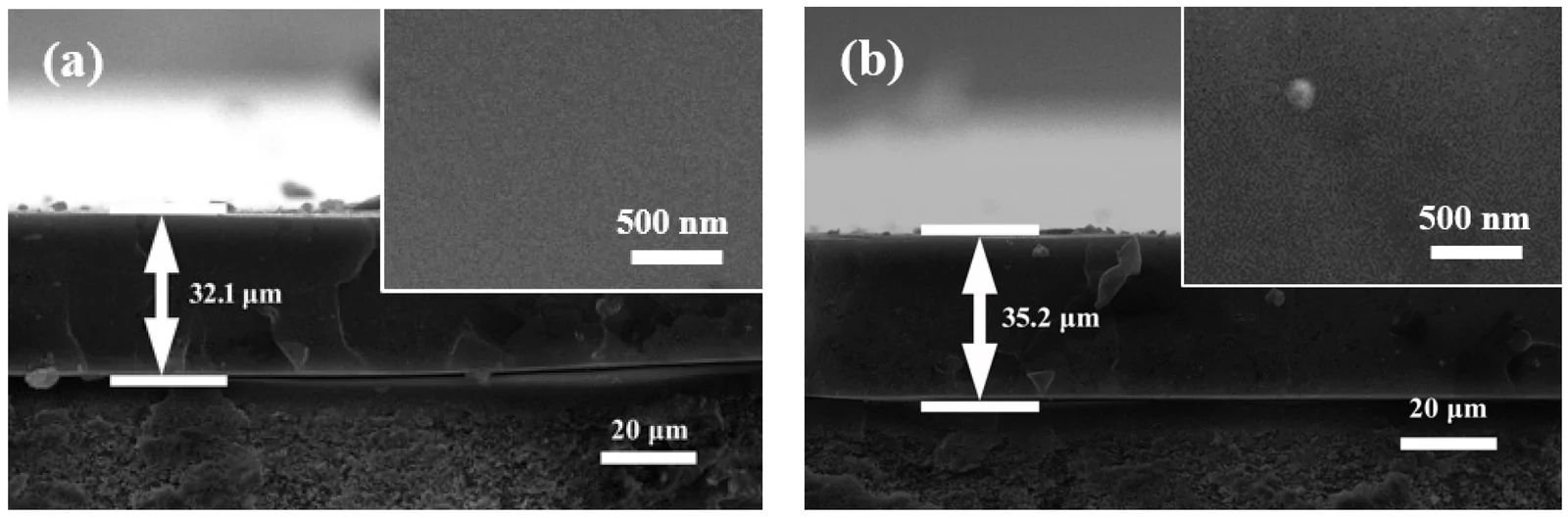

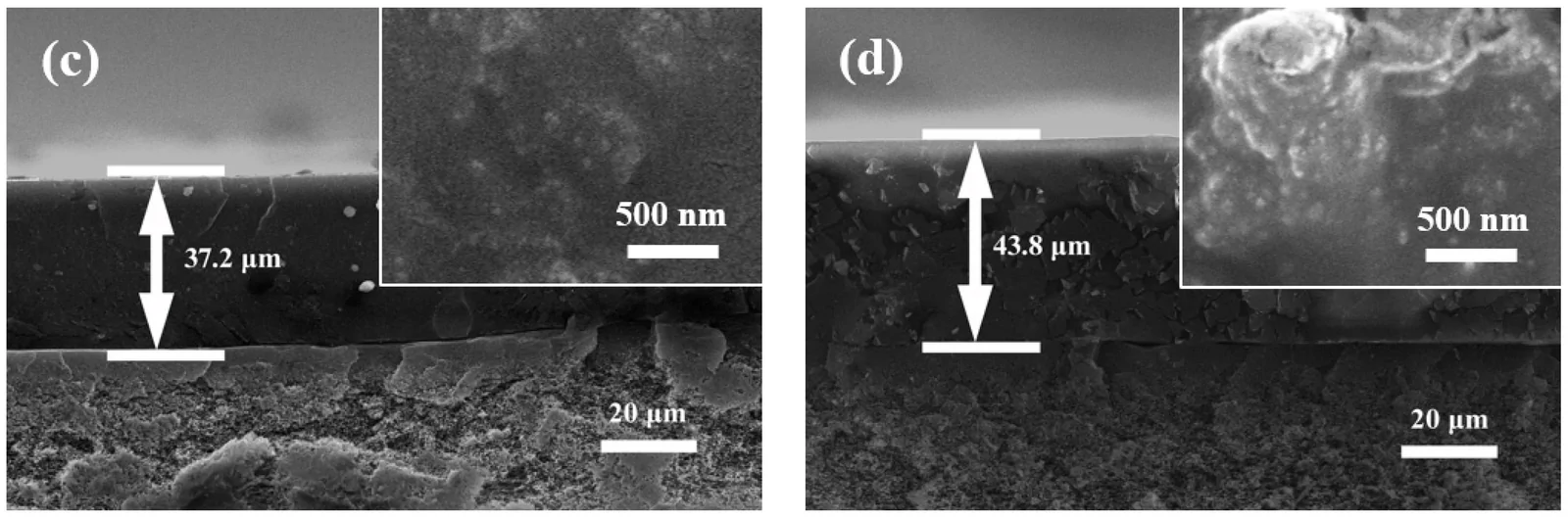
Cross-sectional SEM images of (**a**) the pristine PDMS and PDMS/MIL@MITpBD with MIL@MITpBD loadings of (**b**) 1 wt%, (**c**) 5 wt% and (**d**) 7 wt% MIL@MITpBD (insets in the upper right corner show high-magnification views).

The microstructures of PDMS and PDMS/MIL@MITpBD were observed by SEM, as shown in Figure 4, which shows the cross-sectional images and the corresponding magnified views. The thickness of PDMS is about 32.1 μm, and it increases from 35.2 to 43.8 μm as the MIL@MITpBD loading increases from 1 to 7 wt%. This is mainly because MIL@MITpBD occupies space and thus increases the physical thickness of PDMS/MIL@MITpBD. As shown in the magnified cross-sectional views inserted in the upper right corner of Figure 4a–d, PDMS exhibits a dense and smooth cross-section without obvious interfacial defects. With increased loading of MIL@MITpBD, more filler particles can be observed in the membrane. When the loading reaches 7 wt%, the filler undergoes obvious aggregation and forms interfacial defects in the membrane, leading to a decline in separation performance.

**Figure 5:**
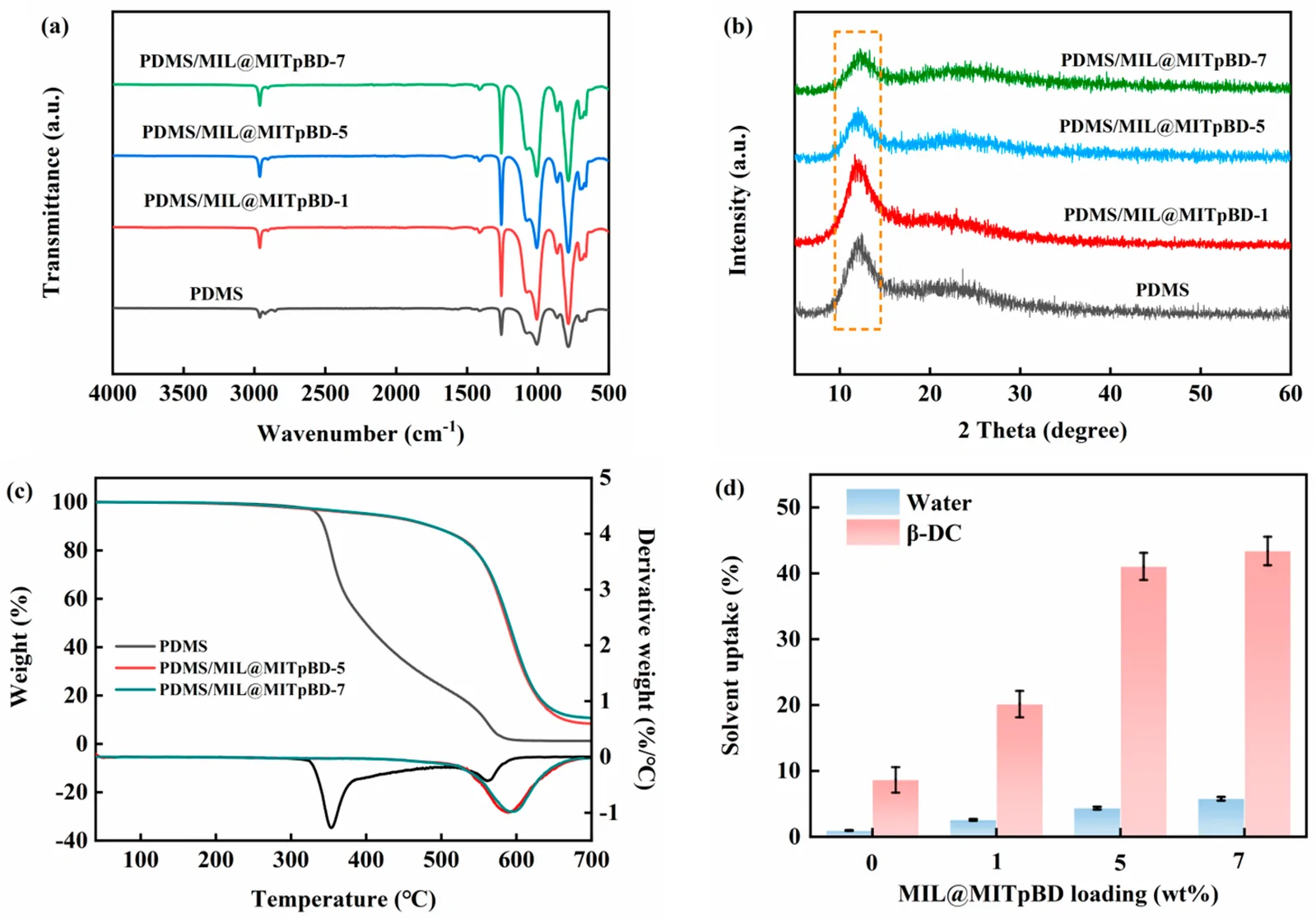
(**a**) ATR−FTIR, (**b**) XRD, (**c**) TGA/DTG, and (**d**) solvent uptake of PDMS/MIL@MITpBD.

As shown in the ATR-FTIR spectra in Figure 5a, PDMS/MIL@MITpBD exhibits a C–H stretching peak at 2964 cm^−1^, corresponding to the –CH_2_– group. The bands at 1409 and 1257 cm^−1^ arise from the asymmetric and symmetric Si–C deformation modes, respectively. Those at 1077 and 1008 cm^−1^ are assigned to the asymmetric and symmetric Si–O–Si stretching modes, while the peaks at 865 and 785 cm^−1^ stem from Si–O and Si–C stretching vibrations, respectively [20]. The spectrum of PDMS/MIL@MITpBD exhibited no detectable characteristic absorption bands attributable to MIL@MITpBD, which is completely encapsulated within the PDMS matrix [21]. The PDMS and PDMS/MIL@MITpBD were characterized by XRD, as shown in Figure 5b; the PDMS displays a broad peak at 11–14°, indicating the amorphous structure of PDMS [22]. In contrast, the diffraction peak intensity of PDMS/MIL@MITpBD decreases with increasing MIL@MITpBD loading, which can be attributed to the uniform dispersion of MIL@MITpBD in PDMS, which disrupts the ordered structure of the polymer chains and restricts their mobility, thereby enhancing the amorphous structures [23].

As illustrated in Figure 5c and Table S3, the mass reduction of PDMS within the range of 281–390 °C corresponds to the thermal breakdown of methyl and silanol moieties. The mass loss between 493 and 607 °C results from chain depolymerization. The *T*_d10_, *T*_onset_, and *T*_max_^a^ are 344 °C, 325 °C, and 353 °C, respectively. After the incorporation of MIL@MITpBD, the thermal stability of PDMS/MIL@MITpBD-5 is significantly enhanced, with *T*_d10_, *T*_onset_, and *T*_max_^a^ increasing to 486 °C, 517 °C, and 578 °C, respectively. This indicates that MIL@MITpBD can significantly improve the thermal stability of MMMs, which is likely due to interfacial interactions (including van der Waals forces and hydrogen bonding) between the polymer matrix and the filler particles, which collectively retard the onset of thermal decomposition [24].

The adsorption properties of PDMS and PDMS/MIL@MITpBD for water and β-DC were tested, as shown in Figure 5d. Due to their inherent hydrophobicity, both of the membranes exhibited low water adsorption but high adsorption affinity for β-DC. As the loading of MIL@MITpBD increased to 7 wt%, the water adsorption rose from 0.96% (PDMS) to 5.78%, while the β-DC adsorption significantly increased from 8.64% (PDMS) to 43.39%. The increasing uptake of H_2_O is primarily attributed to the free volume expansion within PDMS/MIL@MITpBD, whereas the substantial enhancement in β-DC adsorption is ascribed to the abundant imprinted cavities in MIL@MITpBD, which enhance the specific adsorption capacity toward β-DC.

### 3.3. Pervaporation Performance of PDMS/MIL@MITpBD

**Figure 6:**
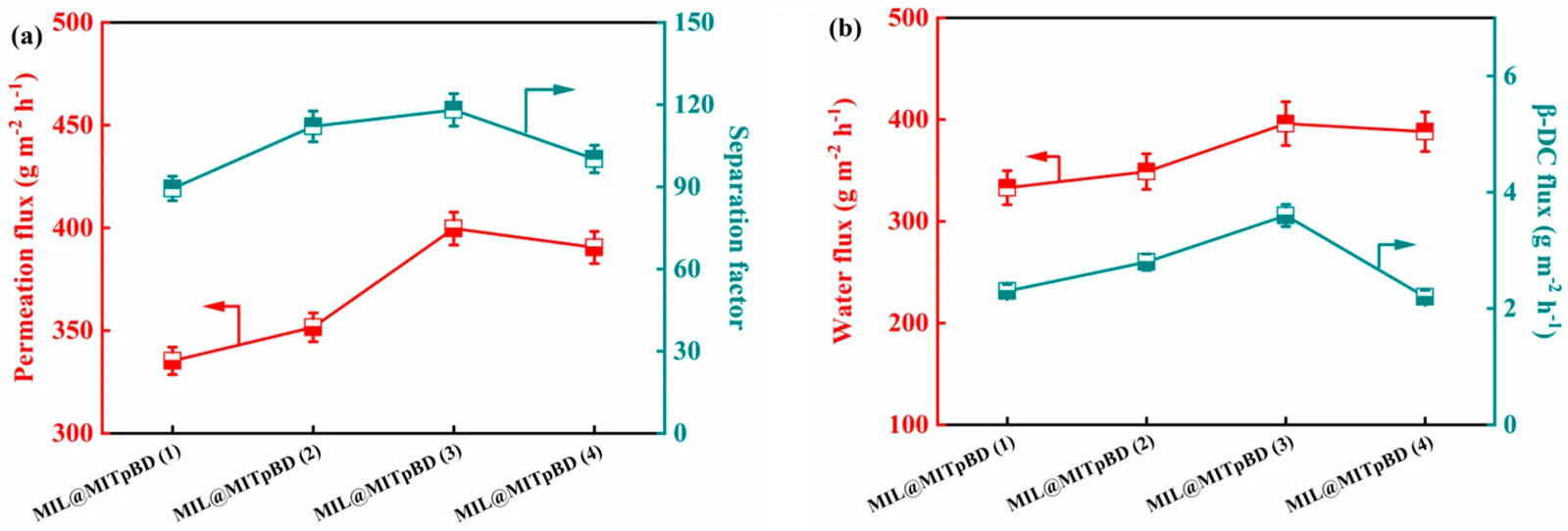
Dependence of (**a**) the *J* and *β*_β-DC/H2O_ and (**b**) *J*_H2O_ and *J*_β-DC_ of PDMS/MIL@MITpBD on the thickness of the imprinted layer.

By varying the amounts of Tp and BD-OH used for TpBD-OH synthesis, the thickness of the imprinted MITpBD shell layer on the surface of the MIL-101 core could be tuned in order to investigate how the imprinted MITpBD shell-layer thickness affects β-DC separation performance. With a fixed MIL@MITpBD loading of 1 wt% and the wet thickness of PDMS/MIL@MITpBD kept at 200 μm, as the thickness of the imprinted MITpBD shell layer coated on MIL-101 increases while X increases from 1 to 4 for PDMS/MIL@MITpBD(X), *J*, *J*_β-DC_, *J*_H2O_, and *β*_β-DC/H2O_ all exhibit a rise–peak–drop trend, as shown in Figure 6a,b. Among them, PDMS/MIL@MITpBD(3) demonstrates the highest separation efficiency, with a *J* of 399.7 g m^−2^ h^−1^ and a *β*_β-DC/H2O_ of 118.1. As the thickness of the imprinted MITpBD shell layer coated on MIL-101 increases, the imprinted network formed by TpBD-OH on the surface of MIL-101 gradually becomes more complete, which facilitates the diffusion and transport of β-DC. Meanwhile, the increased number of hydrogen-bonding sites and imprinted cavities enhances the interactions between the imprinted cavities and β-DC, thereby improving *β*_β-DC/H2O_. However, an excessively thick MITpBD shell layer on MIL-101 in PDMS/MIL@MITpBD(4) prolongs the diffusion path and increases diffusion resistance, leading to a decrease in *J*, *J*_β-DC_, and *J*_H2O_. Further, part of the imprinted cavities becomes encapsulated and compressed with the overgrowth of the MITpBD shell layer on MIL-101, which may cause a reduction in the utilization efficiency of the imprinted cavities and then ultimately result in a decrease in *β*_β-DC/H2O_. The pervaporation performance of PDMS/MIL@MITpBD(3) was comprehensively evaluated by the PSI. As shown in Figure S6, the PSI value of PDMS/MIL@MITpBD(3) reached a maximum of 46,827 g m^−2^ h^−1^.

**Figure 7:**
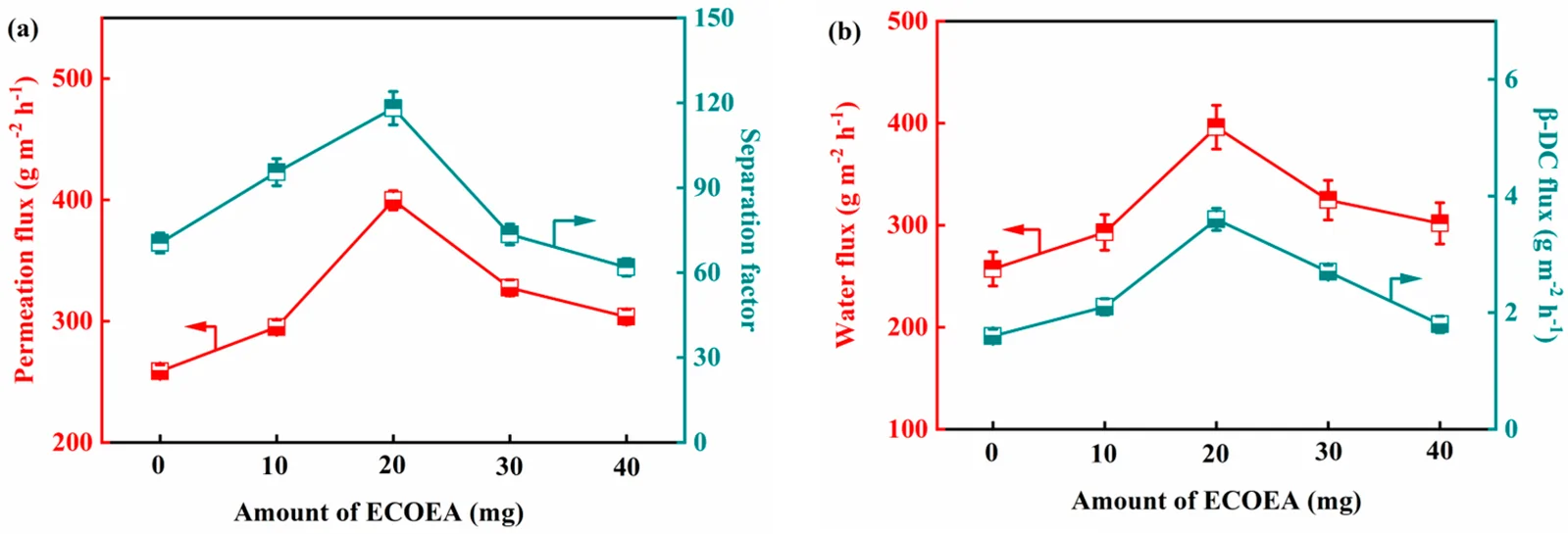
Dependence of (**a**) *J* and *β*_β-DC/H2O_ and (**b**) *J*_H2O_ and *J*_β-DC_ of PDMS/MIL@MITpBD on ECOEA dosage.

Figure 7 illustrates the influence of ECOEA dosage on the β-DC separation performance of PDMS/MIL@MITpBD. When PDMS/MIL@MITpBD(3) was fixed with a fixed MIL@MITpBD loading of 1 wt%, the *J*, *J*_β-DC_, *J*_H2O_ and *β*_β-DC/H2O_ of PDMS/MIL@MITpBD exhibited a rise followed by a drop as ECOEA dosage increased. The optimal separation performance was achieved at an ECOEA dosage of 20 mg, with the corresponding *J* and *β*_β-DC/H2O_ being 399.7 g m^−2^ h^−1^ and 118.1, respectively. As the ECOEA dosage increases, the quantity of imprinted cavities within MIL@MITpBD increases; thereby the specific selectivity of PDMS/MIL@MITpBD toward β-DC is enhanced. Meanwhile, the increased ECOEA dosage also leads to a greater quantity of pores and improved pore connectivity, facilitating the formation of continuous fast mass transfer channels. This reduces the diffusion resistance in PDMS/MIL@MITpBD and enhances the flux of β-DC. However, when the ECOEA dosage exceeded 20 mg, the deeply embedded template molecules became difficult to completely elute. The residual template blocked the porous channels of MIL@MITpBD, reducing the effective diffusion pathways for β-DC and lowering the site utilization, which led to a significant decrease in β-DC permeability (Figure S7a). In addition, an excessive amount of template (ECOEA) caused overcrowding of the template–monomer interactions during the fabrication of the imprinted layer, resulting in the stacking and agglomeration of imprinted cavities and inhibiting the crosslinking polymerization of the imprinted layer. This led to a loose structure, which was unfavorable for β-DC diffusion. At an ECOEA dosage of 20 mg, PDMS/MIL@MITpBD achieved an optimal PSI of 46,827 g m^−2^ h^−1^ (Figure S7b).

**Figure 8:**
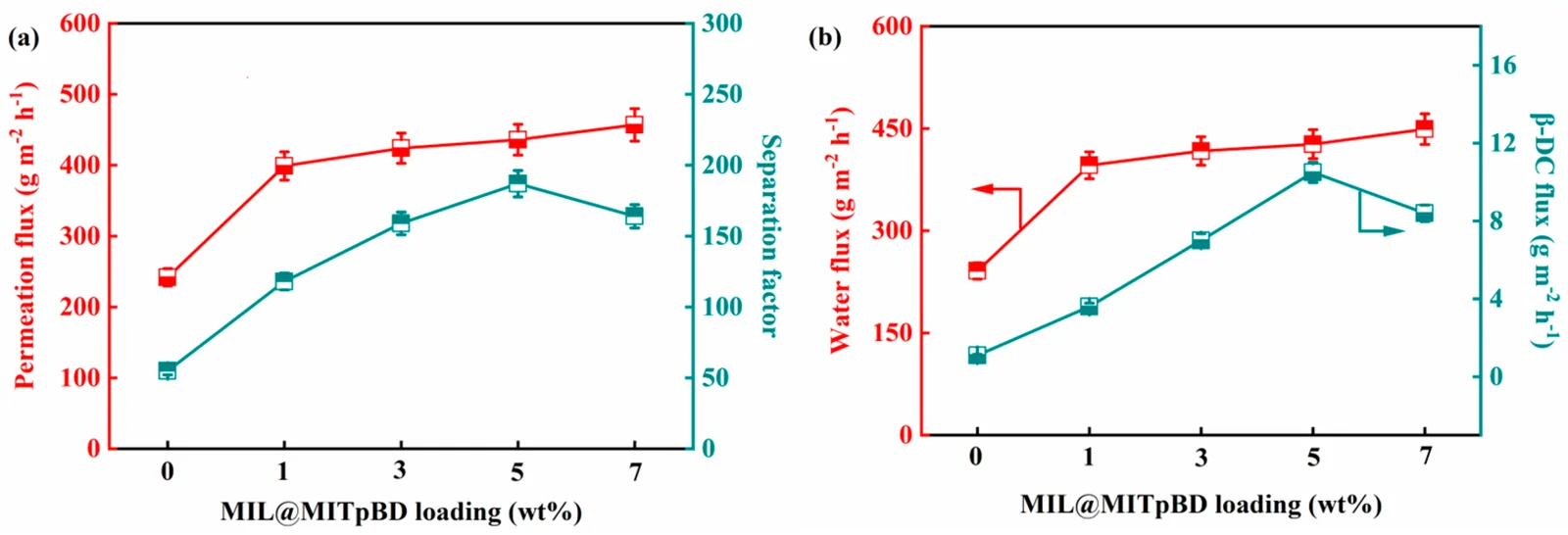
Dependence of (**a**) *J* and *β*_β-DC/H2O_ and (**b**) *J*_H2O_ and *J*_β-DC_ of PDMS/MIL@MITpBD on MIL@MITpBD loading.

As shown in Figure 8, the effect of MIL@MITpBD loading on the separation performance of PDMS/MIL@MITpBD was investigated. With increasing MIL@MITpBD loading in the PDMS/MIL@MITpBD, both *J* and *β*_β-DC/H2O_ exhibited a continuous upward trend. When the loading reached 5 wt%, the PDMS/MIL@MITpBD achieved optimal performance, with *J* and *β*_β-DC/H2O_ values of 436.4 g m^−2^ h^−1^ and 187.9, respectively. Compared with the pure PDMS membrane (242.3 g m^−2^ h^−1^, 55), *J* and *β*_β-DC/H2O_ were increased by 80.1% and 241.6%, respectively. When the MIL@MITpBD loading was below 5 wt%, the quantity of imprinted cavities correspondingly increased as the loading increased. The selective recognition ability for the size and shape enhanced the adsorption of β-DC in the PDMS/MIL@MITpBD. Meanwhile, the imprinted channels within the membrane served as fast diffusion pathways for β-DC. In addition, the increased number of amino and hydroxyl groups strengthens the hydrogen-bonding interactions between the membrane and β-DC, which is in favor of the increasing of *β*_β-DC/H2O_. When the MIL@MITpBD loading was higher than 5 wt%, both *J*_β-DC_ and *β*_β-DC/H2O_ decreased, which is because the MIL@MITpBD dispersed in the membrane poorly and agglomerated. This resulted in the formation of non–selective defects while also reducing the utilization efficiency of the imprinted cavities. Consequently, the selective recognition of β-DC was weakened, leading to a decrease in permeability and selectivity. The weakened selective recognition ability toward β-DC and reduced number of imprinted diffusion channels thereby decreased the *J* and *β*_β-DC/H2O_. A maximum PSI of 81,586 g m^−2^ h^−1^ was recorded for PDMS/MIL@MITpBD-5 and is presented in Figure S8.

**Figure 9:**
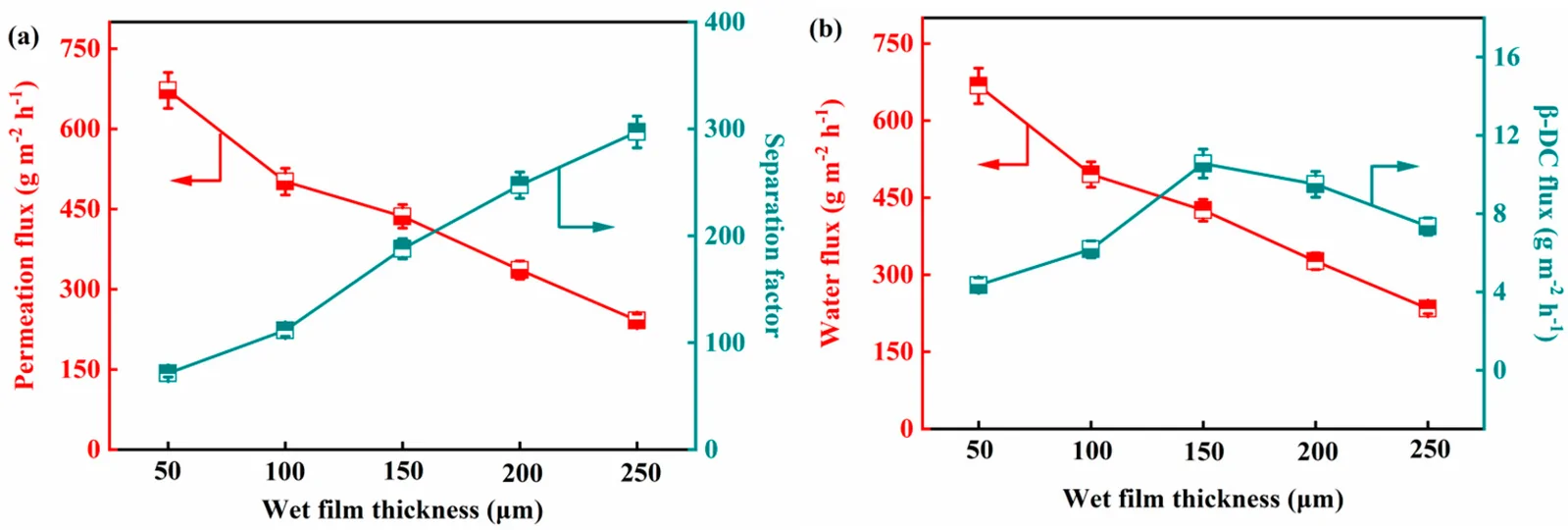
Dependence of (**a**) *J* and *β*_β-DC/H2O_ and (**b**) *J*_H2O_ and *J*_β-DC_ of PDMS/MIL@MITpBD on wet film thickness.

Figure 9a illustrates that the *J* of PDMS/MIL@MITpBD-5 decreased from 672.2 g m^−2^ h^−1^ to 241.8 g m^−2^ h^−1^ with the increase in wet film thickness, while the *β*_β-DC/H2O_ increased from 71.5 to 297.1. An increase in film thickness prolongs the molecular diffusion path and increases diffusion resistance, which is the main reason for the decline in *J*. Inversely, the thickening allows more β-DC molecules to be adsorbed, enhancing the transmembrane driving force and thereby leading to an increase in *β*_β-DC/H2O_. *J*_β-DC_ continuously increased while the membrane thickness was below 150 μm, as shown in Figure 9b, which is because an increase in thickness increases the number of imprinted adsorption sites and imprinted diffusion channels. However, when the film thickness exceeded 150 μm, the extended diffusion path and the repeated stacking of imprinted cavities reduced the diffusion rate of β-DC, leading to a decrease in *J*_β-DC_. As depicted in Figure S9, a maximum PSI value of 82,830 g m^−2^ h^−1^ was achieved at 200 μm.

**Figure 10:**
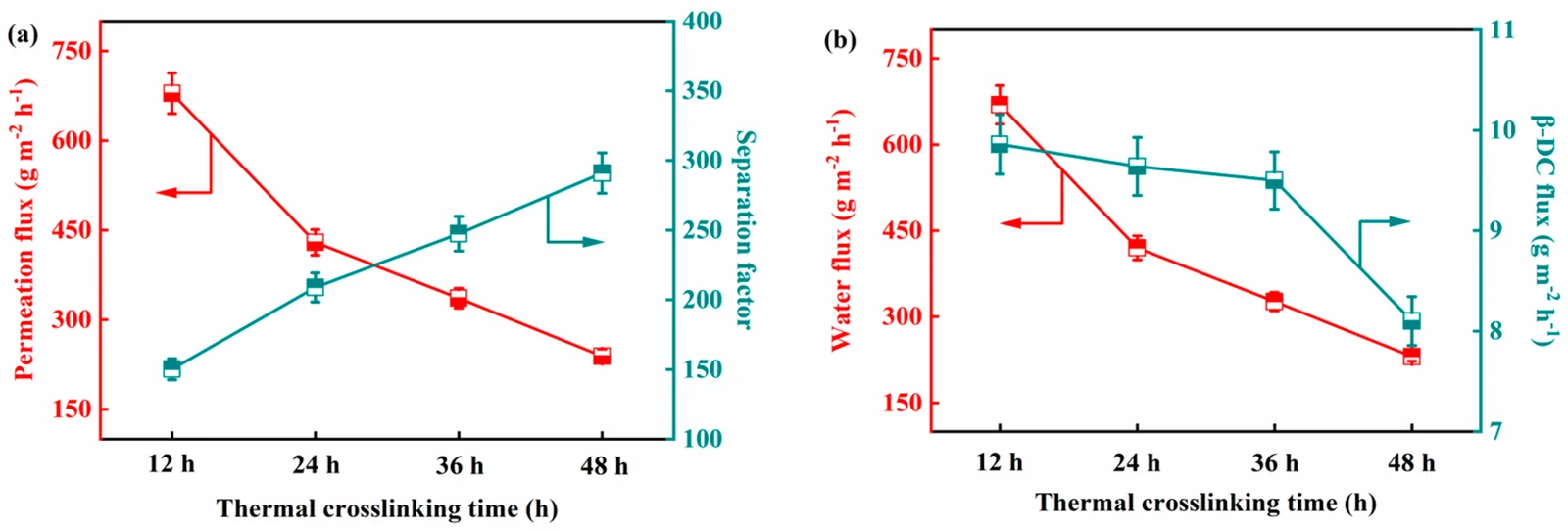
Dependence of (**a**) *J* and *β*_β-DC/H2O_ and (**b**) *J*_H2O_ and *J*_β-DC_ of PDMS/MIL@MITpBD on crosslinking time.

As shown in Figure 10, the *J* of PDMS/MIL@MITpBD-5 decreased from 679.1 g m^−2^ h^−1^ to 238.8 g m^−2^ h^−1^ when crosslinking time increased from 12 h to 48 h, while the *β*_β-DC/H2O_ increased from 150.2 to 291. Prolonging the thermal crosslinking time enhances the crosslinking degree between PDMS and TEOS, resulting in a denser structure at 48 h. The reduced free volume and decreased chain mobility increase the permeation resistance, thereby leading to a decline in *J*, *J*_β-DC_, and *J*_H2O_. Meanwhile, as the crosslinking time increases, a complete Si-O hydrophobic network gradually forms in the PDMS/MIL@MITpBD-5, enhancing the hydrophobicity toward β-DC, which consequently increases the *β*_β-DC/H2O_. Combined with Figure S10, these results show that when the *β*_β-DC/H2O_ was maintained above 250, the *J*_β-DC_, PSI and β-DC permeability all remained at relatively high levels, with values of 9.51 g m^−2^ h^−1^, 82,842 g m^−2^ h^−1^, and 1.65 × 10^9^ Barrer, respectively.

**Figure 11:**
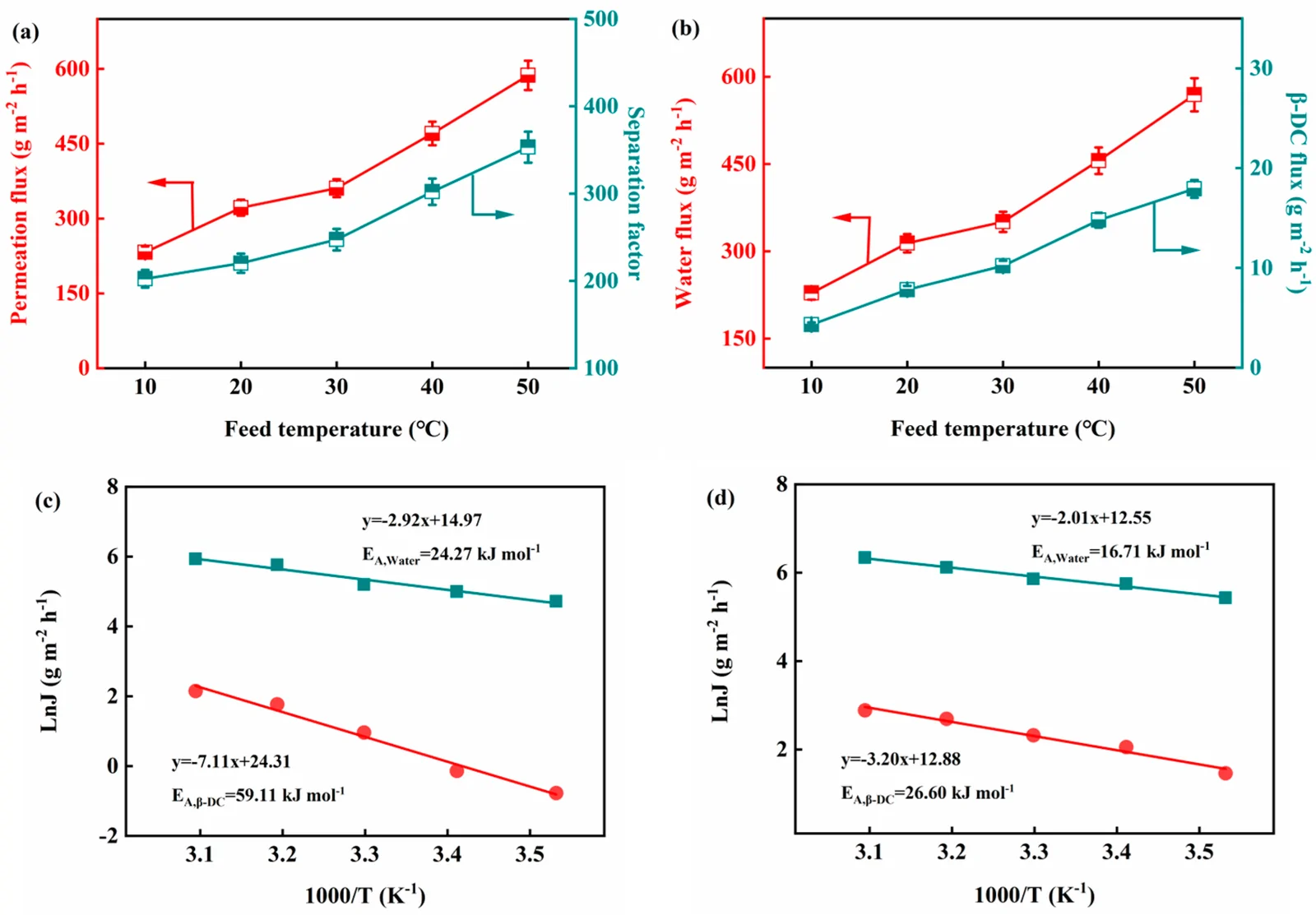
Dependence of (**a**) *J* and *β*_β-DC/H2O_ and (**b**) *J*_H2O_ and *J*_β-DC_ of PDMS/MIL@MITpBD on feed temperature. Arrhenius plots of *J*_β-DC_ and *J*_H2O_ of (**c**) pure PDMS and (**d**) PDMS/MIL@MITpBD vs. 1000/T.

Raising the feed temperature significantly enhances the pervaporation separation performance of PDMS/MIL@MITpBD-5 toward β-DC. In Figure 11a,b, when the feed temperature increases from 10 °C to 50 °C, the permeation flux *J* rises from 232.6 g m^−2^ h^−1^ to 587 g m^−2^ h^−1^, while the separation factor of β-DC/H_2_O increases from 202.5 to 353.2. The effect of feed temperature is essentially attributed to the synergistic reinforcement of thermodynamic and kinetic behaviors in the solution–diffusion mechanism. Elevated temperature intensifies the thermal motion of β-DC and water molecules, enabling them to more readily overcome the mass transfer resistance at the interface between the membrane matrix and the filler, thereby achieving simultaneous enhancement of both flux and selectivity. According to the Arrhenius equation in Equation (S9), the diffusion coefficient increases exponentially with rising temperature. Meanwhile, the elevated temperature raises the vapor partial pressure on the feed side, thereby increasing the driving force across the membrane, which accounts for the increase in flux of the mixed matrix membranes (MMMs) [25]. Moreover, the backbone of PDMS is susceptible to temperature variations; increasing temperature increases the free volume of the polymer membrane and reduces the mass transfer resistance [26]. For PDMS/MIL@TpBD-5, Figure 11d shows that the *E*_a_ of β-DC (26.60 kJ mol^−1^) is higher than that of H_2_O (16.71 kJ mol^−1^), which indicates that the permeation of β-DC is more sensitive to temperature variations. Consequently, the separation factor and selectivity of β-DC/H_2_O are significantly enhanced as temperature increases. The *E*_a_ of β-DC in PDMS/MIL@TpBD-5 (26.60 kJ mol^−1^) in Figure 11d is substantially lower than that in pure PDMS (59.11 kJ mol^−1^) in Figure 11c. This result demonstrates that, compared with pure PDMS, the imprinted structure of MIL@TpBD-5 provides size-matched, low-resistance transport channels within PDMS/MIL@TpBD-5, thereby significantly reducing the energy barrier for β-DC diffusion.

**Figure 12:**
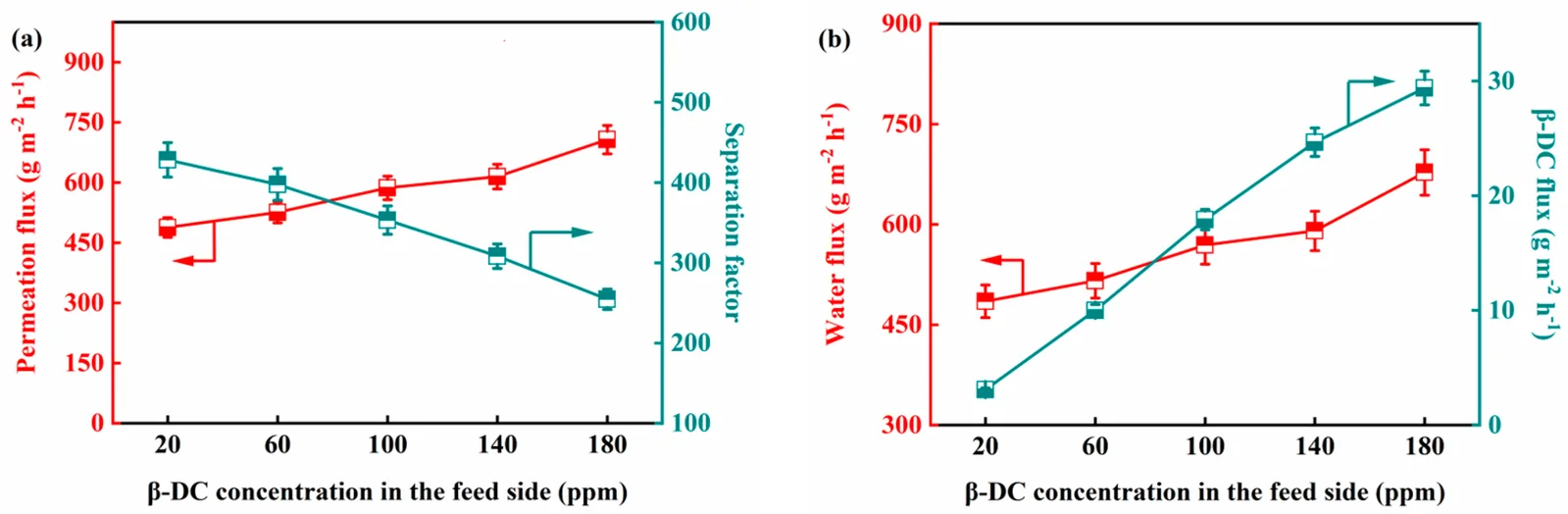
Dependence of (**a**) *J* and *β*_β-DC/H2O_ and (**b**) *J*_H2O_ and *J*_β-DC_ of PDMS/MIL@MITpBD on β-DC concentration.

The effect of feed-side β-DC concentration on the separation performance of PDMS/MIL@MITpBD-5 was investigated, as shown in Figure 12. When the β-DC concentration in the feed solution increased from 20 ppm to 180 ppm, the *J* of PDMS/MIL@MITpBD-5 increased from 488.1 g m^−2^ h^−1^ to 707.1 g m^−2^ h^−1^, while *β*_β-DC/H2O_ decreased from 428.4 to 254.7. Figure 12b further indicates that both *J*_β-DC_ and *J*_H2O_ increased with rising feed concentration. This is attributed to the enhanced concentration gradient on both sides of the membrane and the significantly higher affinity of PDMS/MIL@MITpBD-5 for β-DC than for H_2_O, which together increase the mass transfer driving force. Additionally, the increased adsorption of β-DC induces membrane swelling. This swelling in DMS/MIL@MITpBD-5 increases the free volume, which in turn accelerates the diffusion rates of both components, ultimately leading to an increase in flux. And the increase in the free volume facilitates H_2_O permeation more significantly, resulting in a decline in *β*_β-DC/H2O_. Moreover, membrane swelling causes a decrease in β-DC permeability, as shown in Figure S12a, and the PSI of DMS/MIL@MITpBD-5 also decreases with increasing concentration, as shown in Figure S12b. 

### 3.4. Pervaporation Performance of PDMS/ MIL@MITpBD for Ternary Mixture

**Figure 13:**
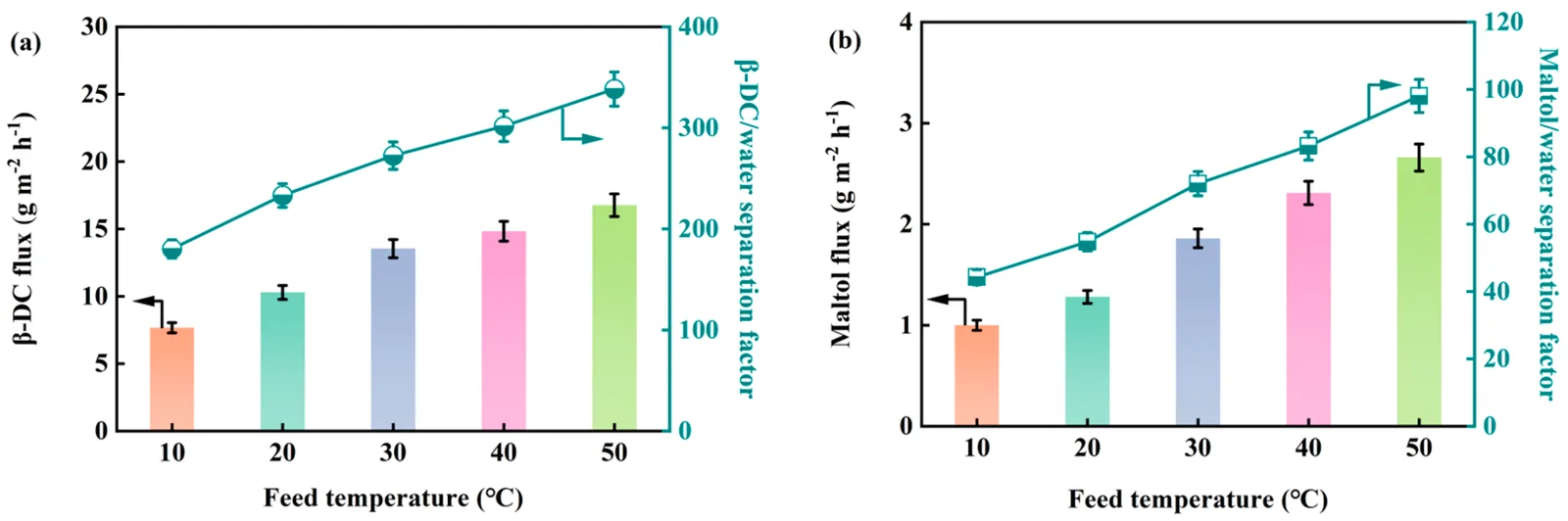
Dependence of (**a**) the *J*_β-DC_ and *β*_β-DC/H2O_ and (**b**) *J*_Maltol_ and *β*_Maltol/H2O_ of PDMS/MIL@MITpBD on maltol concentration in the ternary system.

In practical separation processes with the coexistence of other aromatic compounds, these aromatic components cause competitive permeation with β-DC. Therefore, a ternary β-DC/maltol/H_2_O model solution was constructed, and the effect of different feed temperatures on the separation performance of PDMS/MIL@MITpBD-5 for β-DC was investigated. As depicted in Figure 13, elevating the feed temperature from 10 °C to 50 °C resulted in a progressive enhancement of both *J* and *β*. Specifically, *J*_β-DC_ increased from 7.67 g m^−2^ h^−1^ to 16.77 g m^−2^ h^−1^ (119% improvement), while *J*_Maltol_ increased from 1 g m^−2^ h^−1^ to 2.66 g m^−2^ h^−1^ (166% improvement). Similarly, *β*_β-DC/H2O_ and *β*_Maltol/H2O_ demonstrated significant augmentation, increasing from 180.3 to 338.5 (88% improvement) and from 44.2 to 98.1 (122% improvement), respectively. Increasing the feed temperature enhances the transmembrane driving force for both β-DC and maltol, as well as the free volume of the membrane [27,28]. Furthermore, it also intensifies the thermal motion of molecules, thereby facilitating their diffusion through the membrane. *J*_β-DC_ and *β*_β-DC/H2O_ were significantly higher than *J*_Maltol_ and *β*_Maltol/H2O_, respectively, which can be attributed to the selective recognition of PDMS/MIL@MITpBD-5 for β-DC by the imprinted cavities. Combined with Figure S13, these results show that *E*_a, β-DC_ (14.71 kJ mol^−1^) is lower than *E*_a, maltol_ (19.37 kJ mol^−1^), indicating that β-DC permeates through the membrane more easily than maltol. 

**Figure 14:**
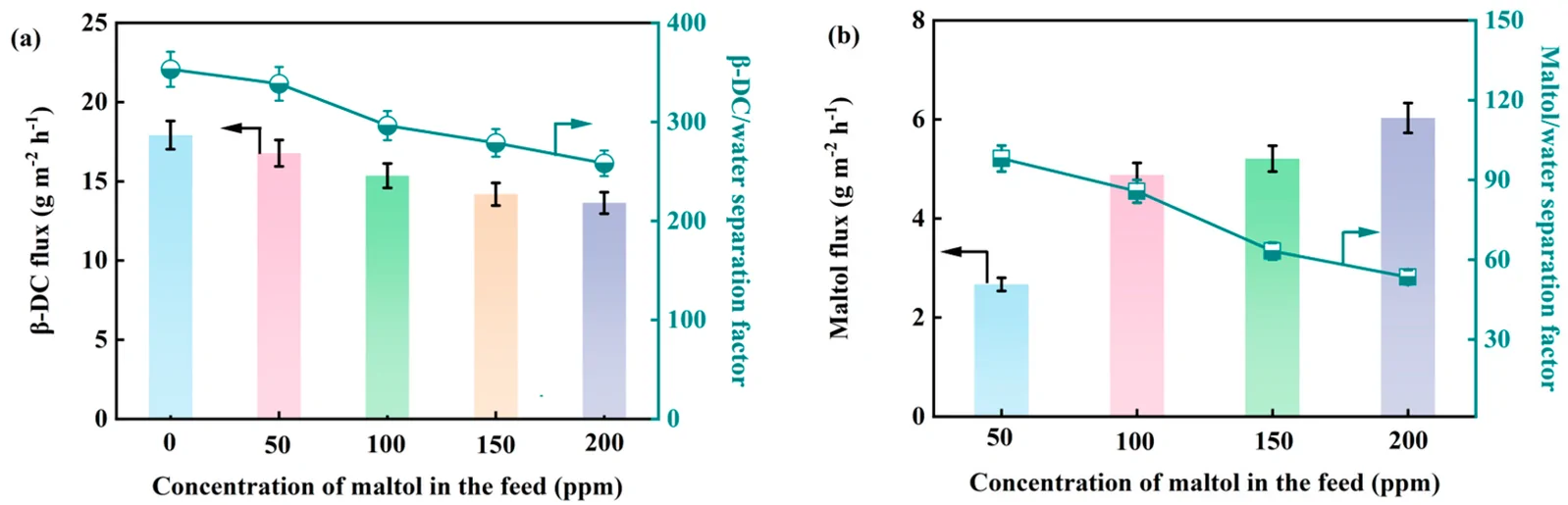
Dependence of (**a**) the *J*_β-DC_ and *β*_β-DC/H2O_ and (**b**) *J*_Maltol_ and *β*_Maltol/H2O_ of PDMS/MIL@MITpBD on maltol concentration in the ternary system.

As shown in Figure 14, when the maltol concentration was increased from 0 to 200 ppm while the β-DC concentration was fixed at 100 ppm, *J*_maltol_ increased to 6.03 g m^−2^ h^−1^, whereas *β*_maltol/H2O_ decreased from 98.08 to 53.48. Regrettably, the presence of maltol significantly inhibited the transport of β-DC: *J*_β-DC_ decreased from 17.92 g m^−2^ h^−1^ to 13.64 g m^−2^ h^−1^, and *β*_β-DC/H2O_ dropped from 353.21 to 258.25. This indicates a direct competitive relationship between β-DC and maltol, primarily attributed to the preferential adsorption of the hydroxyl groups of maltol molecules onto the membrane. Nevertheless, the selective recognition of β-DC by the imprinted cavities in PDMS/MIL@MITpBD-5 is still significant for maintaining a high *β*_β-DC/H2O_ (258.25).

**Figure 15:**
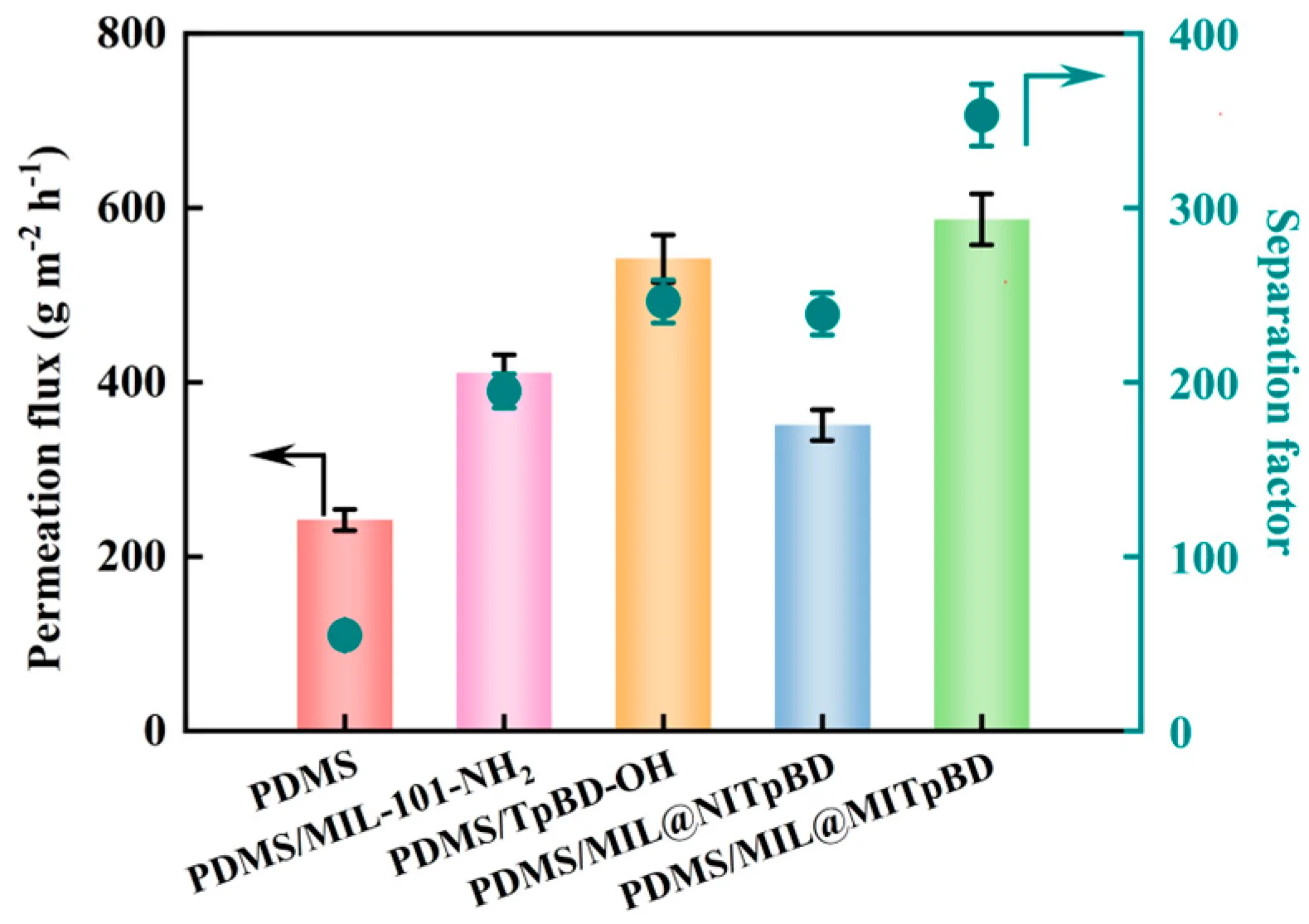
Effect of different fillers on pervaporation performance of MMMs.

To investigate the separation mechanism of the imprinted cavities in PDMS/MIL@MITpBD, PDMS, PDMS/MIL-101-NH_2_, PDMS/TpBD-OH, and PDMS/MIL@NITpBD were fabricated and tested. MIL@NITpBD represents non–imprinted MIL@TpBD with a core–shell structure, and its synthesis details are given in Section S2.4. MIL-101-NH_2_ and TpBD-OH are prepared following the methods described in Sections S2.2 and S2.3, respectively. As shown in Figure 15, both the *J* and *β*_β-DC/H2O_ of MIL-101-NH_2_ (411 g m^−2^ h^−1^, 195) and TpBD-OH (542 g m^−2^ h^−1^, 246) were higher than those of pure PDMS (242 g m^−2^ h^−1^, 55). This improvement can be attributed to the hydrogen-bonding interactions between −NH_2_ (MIL-101-NH_2_)/−OH (TpBD-OH) and C=O (β-DC), as well as the π–π interactions between the benzene ring framework (MIL-101-NH_2_ /TpBD-OH) and C=C (β-DC). However, PDMS/MIL@NITpBD (representing non-imprinted MIL@TpBD without imprinted cavities) exhibited the lowest separation performance (351 g m^−2^ h^−1^, 239) among all MMMs. This may be attributed to the fact that a large number of −NH_2_ groups are encapsulated during the in situ growth of TpBD-OH on the surface of MIL-101-NH_2_, thereby reducing the accessibility of the −NH_2_ sites. On the other hand, the encapsulation process may also block the pores of MIL-101-NH_2_ while simultaneously extending the diffusion pathways of molecules, both of which contribute to the decrease in the *J* and *β*_β-DC/H2O_ of PDMS/MIL@NITpBD. Notably, the *J* and *β*_β-DC/H2O_ of PDMS/MIL@MITpBD with imprinted cavities (587 g m^−2^ h^−1^, 353) were higher than those of the non-imprinted PDMS/MIL@NITpBD, which is because the imprinted cavities enhance the selective recognition of β-DC by virtue of size, shape, and hydroxyl functional group interactions.

**Scheme 2:**
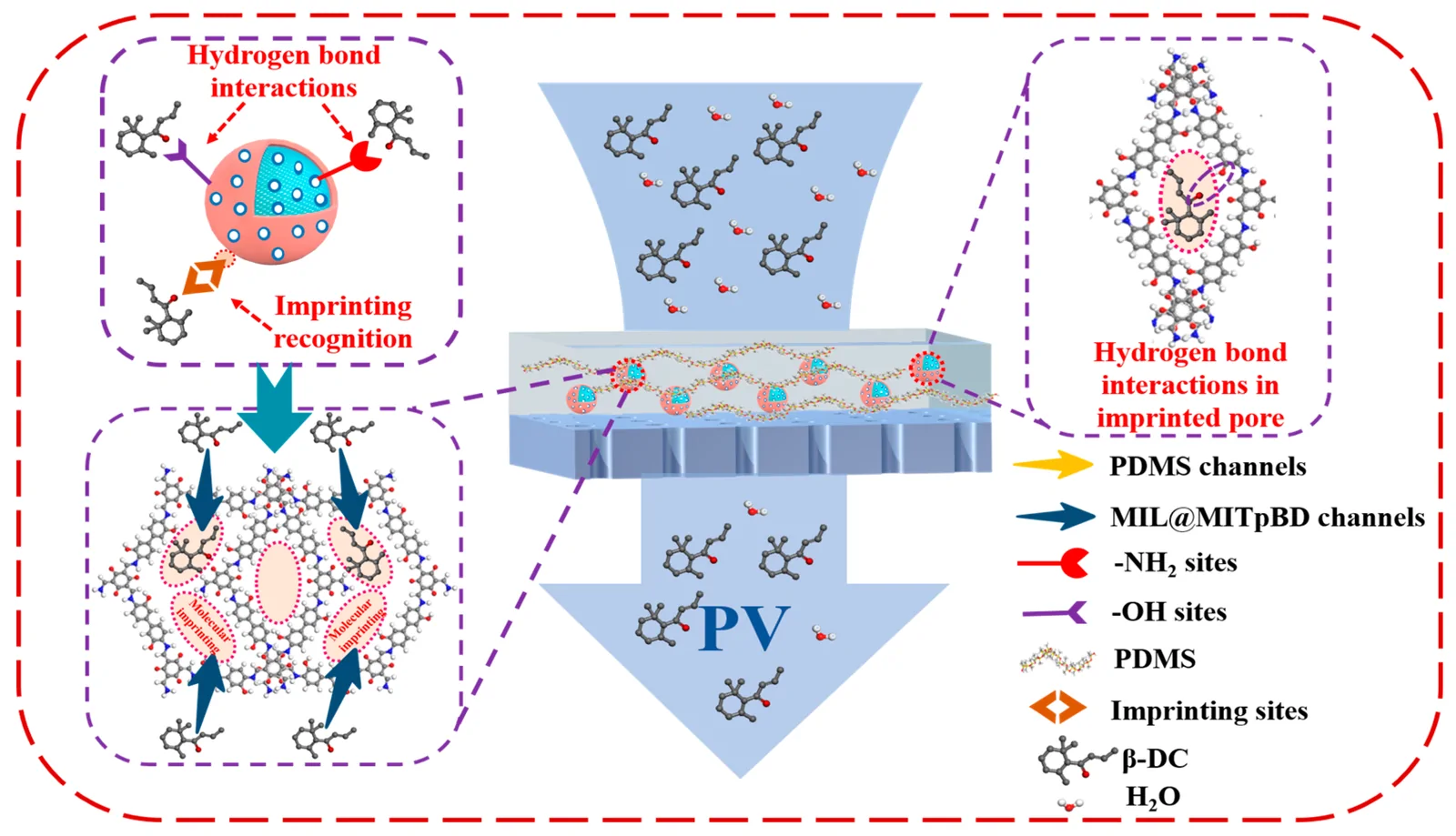
Schematic diagram of the separation mechanism of the PDMS/MIL@MITpBD MMMs.

A schematic illustration of the separation mechanism of β-DC in PDMS/MIL@MITpBD is shown in Scheme 2. The mass transfer of β-DC in PDMS/MIL@MITpBD follows the solution–diffusion mechanism. The transport pathways of β-DC within the membrane can be divided into two types: the slow transport channels through the PDMS matrix and the fast transport channels through MIL@MITpBD. The inter-chain transport is driven by the chemical potential gradient, which promotes the rapid permeation of β-DC. However, due to the restriction of relatively dense PDMS matrix, the mass transfer rate through this pathway is significantly slower. The imprinted MIL@MITpBD is the key to enhancing the separation performance. During the transport process, β-DC molecules are specifically attracted by the imprinted cavities in terms of size and shape, thereby being adsorbed and further transported. Moreover, the imprinted cavities in MIL@MITpBD contain abundant −OH groups, which can form hydrogen-bonding interactions with β-DC. Secondarily, the transport of β-DC within the channels composed of the imprinted cavities is also facilitated by hydrogen-bonding interactions with −NH_2_ groups of MIL-101-NH_2_. Under the synergistic effect of the imprinted cavities and the hydrogen-bonding active sites, β-DC molecules are preferentially adsorbed and transported, thereby effectively improving the separation performance of PDMS/MIL@MITpBD.

### 3.5. Long-Term Stability Test of PDMS/MIL@MITpBD

**Figure 16:**
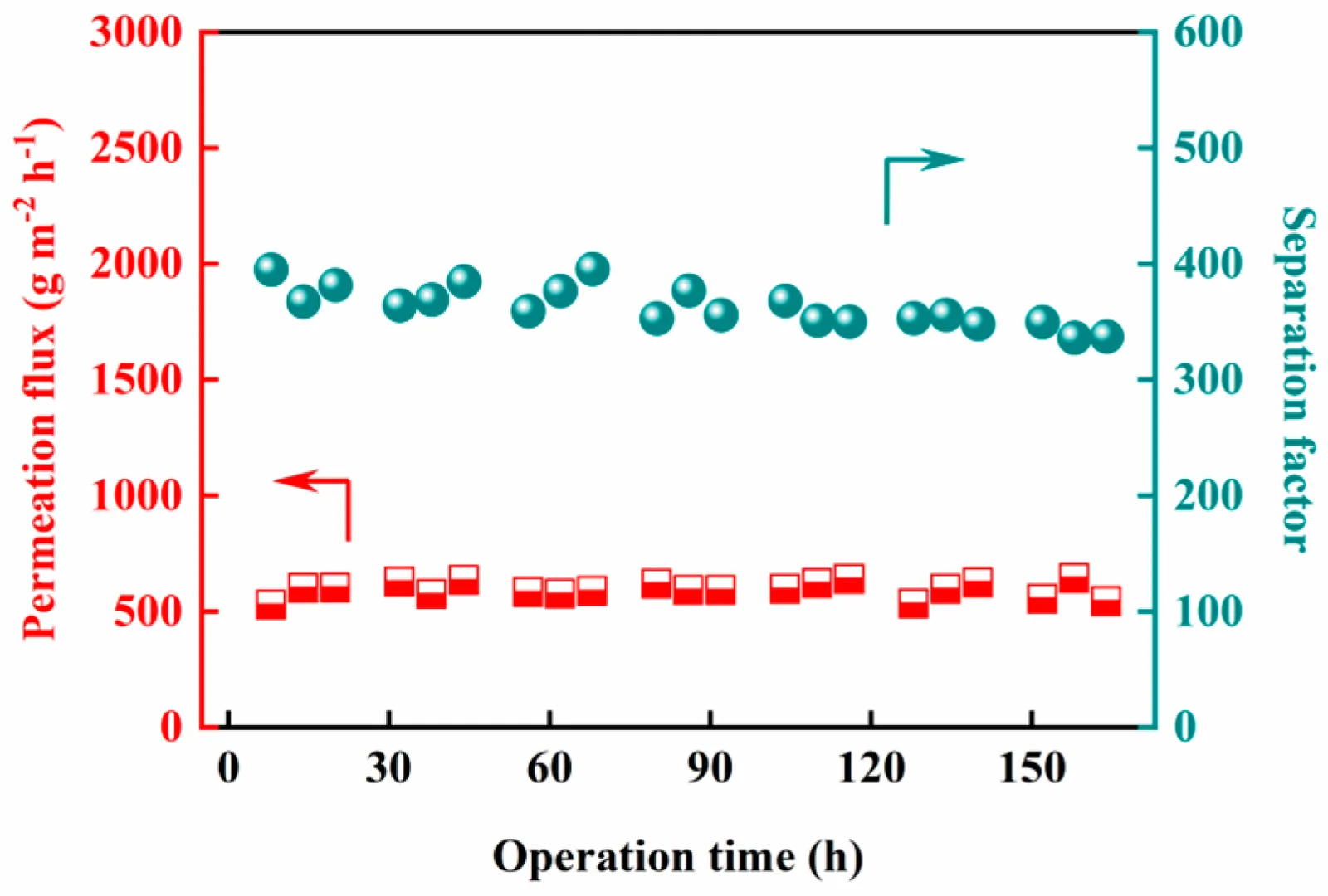
Stability test of PDMS/MIL@MITpBD.

The pervaporation stability of PDMS/MIL@MITpBD-5 was evaluated over a period of 168 h at an operating temperature of 50 °C and a β-DC concentration of 100 ppm. During the whole test, the feed solution remained in continuous contact with the surface of the PDMS/MIL@MITpBD-5, and the β-DC was replenished in time to maintain the concentration of 100 ppm. As shown in Figure 16, the *J* and *β*_β-DC/H2O_ of PDMS/MIL@MITpBD-5 exhibited good stability throughout the 168 h. *β*_β-DC/H2O_ decreased slightly to 341 and *J* increased slightly to 593.8 g m^−2^ h^−1^ at 134 h, which could be attributed to membrane swelling caused by prolonged contact with the feed solution, indicating that the imprinted PDMS/MIL@MITpBD-5 has promising application prospects for the separation of β-DC.

## 4. Conclusions

In this study, a molecularly imprinted material with a core–shell structure of MIL-101-NH_2_ as the core and TpBD-OH as the shell, MIL@MITpBD, was successfully constructed and then incorporated into PDMS to prepare MMMs. The resulting PDMS/MIL@MITpBD was used for β-damascone (β-DC) separation by pervaporation. The experimental results showed that, at 50 °C (operation temperature) and 100 ppm (feed concentration), the *J* and *β*_β-DC/H2O_ of PDMS/MIL@MITpBD reached 587 g m^−2^ h^−1^ and 353.2, respectively, demonstrating excellent pervaporation performance along with good long-term operational stability. The outstanding separation efficiency originated from the cooperative synergy of the selective recognition of imprinted cavities and the well–ordered mass transfer channels. This work provides an effective strategy for membrane design for pervaporation of trace organic compounds, showing broad application prospects in the efficient separation of thermally sensitive and low–concentration organics such as β-DC.

## Data Availability

The raw data supporting the conclusions of this article will be made available by the authors on request.

## Supplementary Materials

The following supporting information can be downloaded at: https://www.mdpi.com/article/10.3390/membranes16100347/s1, The supporting information can be found at the Supplementary Materials [29,30]. Figure S1. The chemical structures of (a) β-DC and (b) ECOEA; Figure S2. UV-Vis absorption spectra (a) and calibration curve (b) of β-DC; Figure S3. FTIR spectra of ECOEA, MIL@NITpBD, MIL@TpBD-ECOEA and MIL@MITpBD; Figure S4. SEM images of (a,b) MIL-101-NH2(Cr), (c,d) TpBD-OH, (e,f) MIL@TpBD-ECOEA and (g,h) MIL@MITpBD; Figure S5. Cross-sectional SEM images of the PSf substrate near its surface; Figure S6. Impact of the thickness of the imprinted layer on (a) P*_β-DC_* and α*_β-DC/H2O_*, (b) PSI; Figure S7. Impact of ECOEA dosage on (a) P*_β-DC_* and α*_β-DC/H2O_*, (b) *PSI*; Figure S8. Impact of MIL@MITpBD loading on (a) P*_β-DC_* and α*_β-DC/H2O_*, (b) *PSI*; Figure S9. Impact of wet film thickness on (a) P*_β-DC_* and α*_β-DC/H2O_*, (b) *PSI*; Figure S10. Impact of crosslinking time on (a) P*_β-DC_* and α*_β-DC/H2O_*, (b) *PSI*; Figure S11. Impact of feed temperature on (a) P*_β-DC_* and α*_β-DC/H2O_*, (b) *PSI*; Figure S12. Impact of β-DC concentration on (a) P*_β-DC_* and α*_β-DC/H2O_*, (b) *PSI*; Figure S13. In ternary system, Arrhenius plots of *J*_β-DC_, *J*_Maltol_ and *J*_H2O_ in PDMS/MIL@MITpBD vs. 1000/T; Table S1. The pore size distribution parameters of MIL-101-NH2, TpBD-OH, MIL@TpBD-ECOEA, MIL@NITpBD and MIL@MITpBD; Table S2. The characteristic parameters of MIL-101-NH2, TpBD-OH, MIL@NITpBD and MIL@MITpBD; Table S3. The characteristic parameters of PDMS and PDMS/MIL@MITpBD.
