# Supplementary material for: Molecularly Imprinted PDMS/MIL@MITpBD Mixed Matrix Membranes for β-Damascone Separation
Source: Membranes (Basel). 2026 Oct 19;16(10):347. doi: 10.3390/membranes16100347 (PMC13707310; doi:10.3390/membranes16100347)
Supplement: Supplementary file 1 [file membranes-16-00347-s001.zip › membranes-4603975-supplementary.pdf]

Supplementary Materials

S2.1 Materials

2-Aminoterephthalic acid (2-ATA, 99.9%), 3,3'-dihydroxybenzidine (BD-OH, 99.9%), 1,3,5-triformylphloroglucinol (Tp, 99.9%), (E)-3-(4-chlorobenzoyl) acrylic acid (ECOEA, 99.9%) and β-damascone (β-DC, 99.9%) were purchased from Shanghai Bide Pharmaceutical Technology Co., Ltd. 1,3,5-Trimethylbenzene was purchased from in Shanghai McLean Biochemical Technology Co., Ltd. Polydimethylsiloxane (PDMS, 5000 mPa·s) and tetraethyl orthosilicate (TEOS) were purchased from Jinan Xingfeilong Chemical Co., Ltd. Dibutyltin dilaurate (DBTDL) was purchased from Chengdu Kelong Chemical Reagent Factory. Polysulfone support (PSF, Da = 45000) was purchased from Shandong Jiuzhang Membrane Technology Co., Ltd. 1,4-Dioxane, *n*-hexane (C_6H_{14}, 98%), ethanol (C_2H_5OH, 99.7%) and methanol (CH_3OH, 99.7%) were purchased from Tianjin Tianli Chemical Reagent Co., Ltd. Deionized water (DI) was homemade by Taiyuan University of Technology.

S2.2 Synthesis of MIL-101-NH_2

MIL-101-NH_2 was synthesized according to a previously reported procedure [29]. Briefly, 1.2 g of $Cr(NO_3)_3·9H_2O$ and 0.54 g of 2-ATA were dispersed in 30 mL of deionized water and dissolved under ultrasonication for 30 min. Then, 0.3 g of NaOH was added, and the mixture was transferred to a Teflon-lined autoclave and reacted at 150 °C for 8 h. After the reaction, the product was washed three times with DMF and anhydrous ethanol, followed by vacuum drying at 80 °C to collect MIL-101-NH_2 powder.

S2.3 Synthesis of TpBD-OH

TpBD-OH was synthesized according to a previously reported procedure [19]. Briefly, 32.4 mg of BD-OH (3,3'-dihydroxybenzidine) and 21 mg of Tp (1,3,5-triformylphloroglucinol) were added to a Schlenk tube containing 4 mL of a 1,3,5-trimethylbenzene/1,4-dioxane mixed solvent (v/v, 2 mL/2 mL) and ultrasonically dispersed for 10 min. Then, 0.4 mL of aqueous HAc (6 M) was added. After degassing via three freeze-pump-thaw cycles, the tube was sealed and heated at 120 °C for 72 h. After cooling, the product was washed several times with DMF and methanol, followed by vacuum drying at 80 °C to afford TpBD-OH powder.

S2.4 Synthesis of MIL@NITpBD

MIL@NITpBD (representing non-imprinted MIL@TpBD), was synthesized following the same procedure as MIL@MITpBD in Section 2.2 of the manuscript, except that the template molecule ECOEA was not introduced. Briefly, 32.4 mg of BD-OH was added to 4 mL of a 1,3,5-trimethylbenzene/1,4-dioxane mixed solvent (v/v, 1:1) and stirred for 30 min. Then, 20 mg of MIL-101-NH_2(Cr) and 21 mg of Tp (1,3,5-triformylphloroglucinol) were added. The dispersion was transferred to a Schlenk tube and ultrasonically dispersed for 10 min, followed by the addition of 0.4 mL of aqueous HAc (6 mol/L). After degassing via three freeze-pump-thaw cycles, the tube was sealed and heated at 120 °C for 72 h. After cooling, the product was washed three times with DMF and methanol, followed by vacuum drying at 80 °C to afford MIL@NITpBD.

S2.5 PDMS membrane preparation

The pristine PDMS membrane was prepared using a solution casting-crosslinking method. Initially, 1 g of PDMS was incorporated into 1.75 g of n-hexane and

magnetically stirred at room temperature for 3 h to ensure complete dissolution. Subsequently, a crosslinking agent solution, containing 0.1666 g of TEOS and 0.0167 g of DBTDL in 0.5 g of n-hexane, was added to the PDMS solution. The mixture was stirred at 30 °C for approximately 2 h to achieve homogeneity. When the viscosity was appropriate for the casting, the solution was uniformly coated onto a hydrophobic PSF ultrafiltration support. The coated support was then transferred to an oven and cured at 80 °C for 36 h to complete the thermal crosslinking, yielding the final PDMS membrane.

S2.6 Pervaporation experiment and related formulas

Pervaporation experiments were performed using a laboratory-built setup. The effective membrane area in the stainless-steel membrane module was 8.04 cm^2. The feed solution was circulated at 90 L h^{-1} by a magnetic pump (MP-20RZM, Shanghai Xinxishan Industrial Co., Ltd.), and the pressure on the permeate side was maintained below 300 Pa by a vacuum pump (VRD-8, Zhejiang Feiyue Mechanical & Electrical Co., Ltd.). Both the β-DC concentration and the operating temperature in the feed were adjustable. The concentrations of β-DC in the feed and permeate were determined by a GENESYS 180 UV-Vis spectrophotometer (Thermo Fisher Scientific Co., Ltd.), measuring the absorbance at 232 nm.

The two most critical parameters for evaluating pervaporation performance are the total flux (J) and the separation factor (β). The total flux (J) is defined as the mass of permeate collected per unit time per unit membrane area. J represents the membrane's permeation rate and is calculated by Eq. (S1):

$$J=\frac{W}{A\times t} \quad \text{(S1)}$$

where J is the total flux (g m^{-2} h^{-1}), W is the mass of the permeate (g), A is the effective membrane area (m^2), and t is the operation time (h).

The separation factor (β) quantifies the membrane's ability to separate target components. A higher value of β corresponds to a more pronounced separation effect. It is defined by Eq. (S2):

$$\beta_{i/j}=\frac{y_i/y_j}{x_i/x_j} \tag{S2}$$

where β is the separation factor (dimensionless), y_i and y_j are the mass fractions of components i and j in the permeate side, and x_i and x_j are the corresponding mass fractions in the feed side.

The fractional flux (J_i) of component i represents the mass of component i passing through a unit membrane area per unit time, which is defined as in Eq. (S3):

$$J_i=J\times y_i \tag{S3}$$

where J_i is the partial flux of component i (g m^{-2} h^{-1}); J is the total permeate flux (g m^{-2} h^{-1}); and y_i is the mass fraction of component i in the permeate.

Membrane permeability (P_i, Barrer) and selectivity ($\alpha_{i/j}$) are defined according to Eqs. (4-6):

$$P_i=10^{-6}\times\frac{J_i\times L}{\Delta p_i} \tag{S4}$$

$$\Delta p_i=X_i\gamma_i P_i^{sat}-Y_i P^P \tag{S5}$$

$$\alpha_{i/j}=\frac{P_i}{P_j} \tag{S6}$$

where L (μm) denotes the thickness of the membrane selective layer; Δp_i (Pa) represents the pressure difference across the membrane. X_i denotes the molar fraction

of component i on the feedstock side; Y_i represents as the molar fraction of component i on the permeate side; γ_i is the activity coefficient of component i calculated under the UNIQUAC model using Aspen V11; P^{sat}_i (Pa) denotes the saturated vapor pressure of component i under the UNIQUAC model; P^P denotes the pressure on the permeation side (0 Pa); and P_j denotes the permeability of component j.

Due to the well-known trade-off effect in pervaporation, membranes with high flux tend to possess a low separation factor, and conversely. Hence, the pervaporation separation index (*PSI*), defined in Eq. (S7), is thus employed to express the overall separation capability of the membrane:

$$PSI=J\times(\beta-1) \tag{S7}$$

where J denotes the permeate flux of the membrane, (g m^{-2} h^{-1}); and β denotes the separation factor of the pervaporation membrane.

S2.7 Characterization methods

The surface morphologies of MIL@MITpBD and PDMS/MIL@MITpBD were observed by scanning electron microscopy (SEM, Hitachi SU8010, Japan). Infrared spectroscopy of the samples was recorded using an ALPHA II spectrometer (FTIR, Bruker, Germany) within the wavenumber range of 400-4000 cm^{-1} at a resolution of 4 cm^{-1}. X-ray diffraction (XRD, Shimadzu XRD-6000, Japan) was employed to characterize the crystal structure of the fillers and membranes, with a 2θ range of 5-70° and a scanning speed of 5 ° min^{-1}. X-ray photoelectron spectroscopy (XPS, Thermo Fisher ESCALAB Xi+, USA) was used for the characterization of MIL@MITpBD. The BET surface area and porosity of the fillers were tested by a V-Sorb X800 surface area

and porosity analyzer (CIQTEK, China) at 77 K.

S2.8 Solvent uptake test

The adsorption characteristics of the membranes towards β-DC and water were evaluated using a gravimetric method following a literature procedure [30]. First, membrane samples (2 cm × 2 cm) were dried in an oven for 8 h and weighed to obtain the dry weight (W_D, g). Subsequently, the samples were immersed separately in pure β-DC and water for 48 h at room temperature to reach sorption equilibrium. After immersion, each sample was removed, quickly wiped with filter paper to remove surface liquid, and immediately weighed to obtain the swollen weight (W_S, g). Each measurement was performed in triplicate, and the average value was used. The degree of adsorption for each liquid is calculated using the following Eq. (S8):

$$\text{Solvent uptake (\%)}=\frac{W_S\text{-}W_D}{W_D}\times 100 \quad \text{(S8)}$$

S2.9 Arrhenius equation

The dependence of permeation flux on temperature is further investigated using the Arrhenius equation (Eq. (S9)):

$$\mathrm{Ln}J_i=\mathrm{Ln}J_0-\frac{E_a}{R\times T} \quad \text{(S9)}$$

where J_i is the permeation flux of component i, (g m^{-2} h^{-1}); J_0 is the preexponential factor; E_a is the apparent activation energy (kJ mol^{-1}); R is the gas constant (J mol^{-1} K^{-1}), and T is the temperature of the feedstock liquid (K).

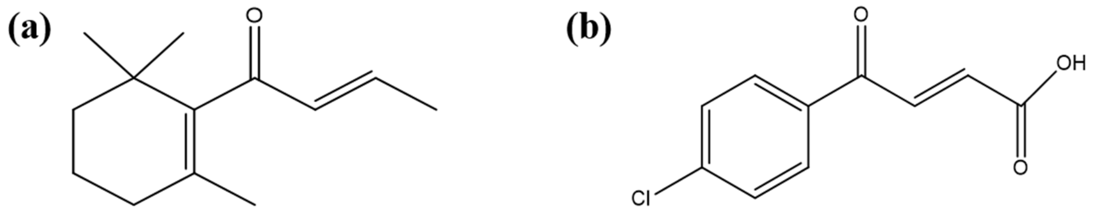

Fig. S1 The chemical structures of (a) β-DC and (b) ECOEA.

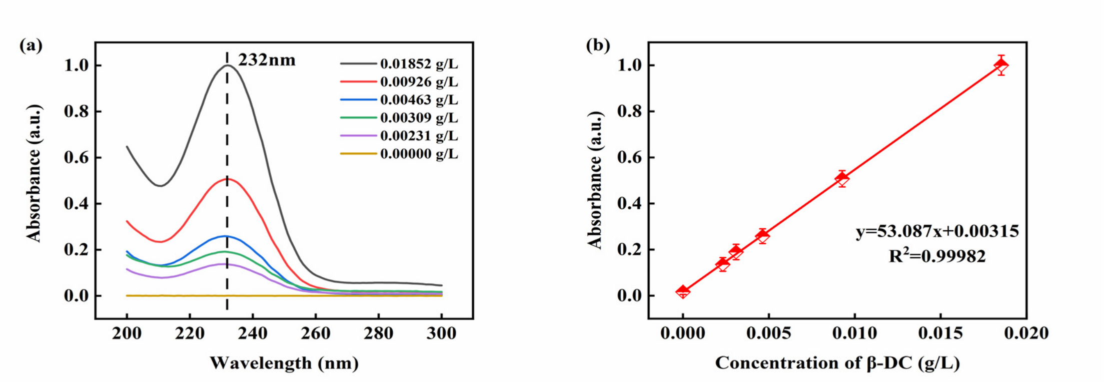

Fig. S2 UV-Vis absorption spectra (a) and calibration curve (b) of β-DC.

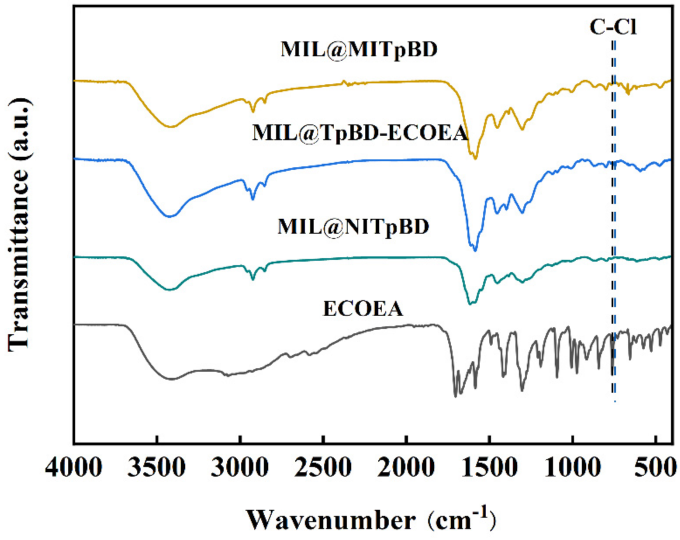

Fig. S3 FTIR spectra of ECOEA, MIL@NITpBD, MIL@TpBD-ECOEA and MIL@MITpBD

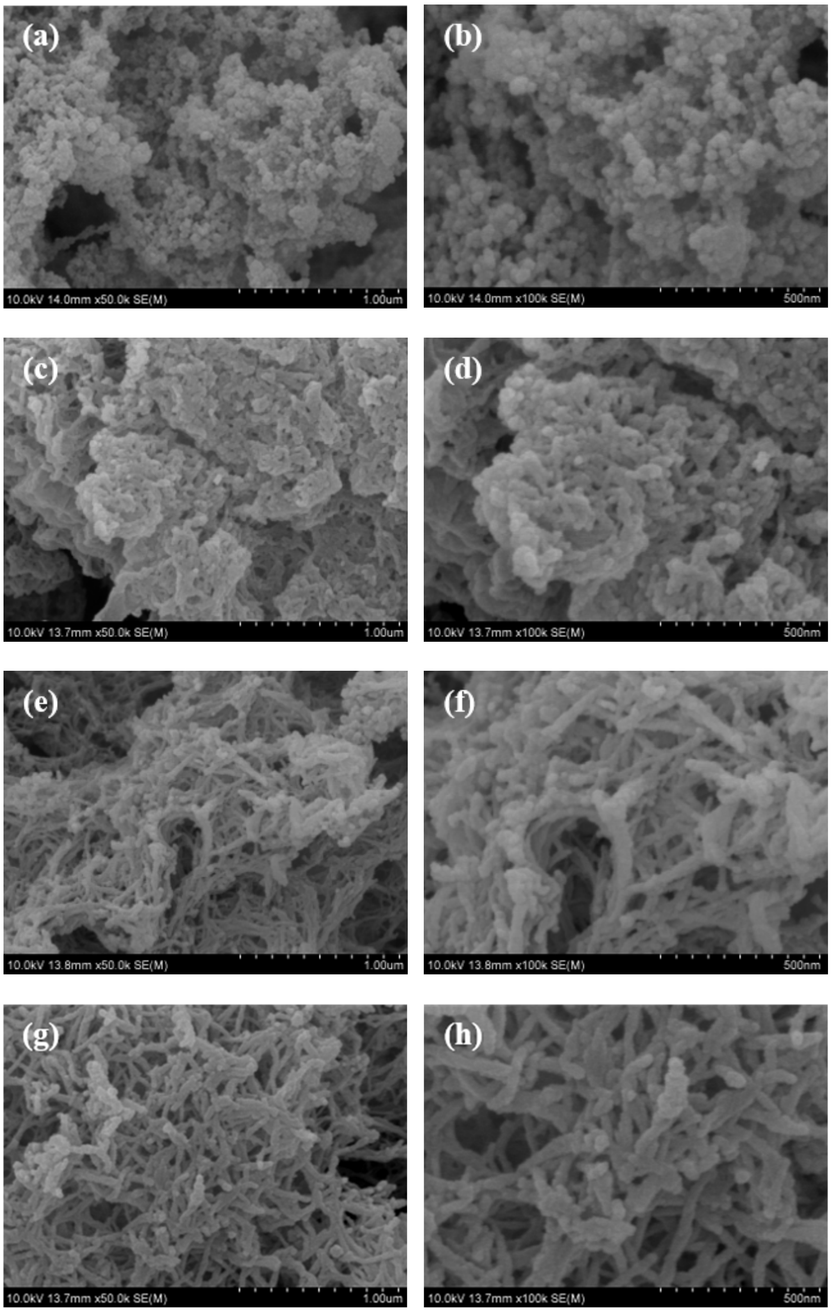

Fig. S4 SEM images of (a, b) MIL-101-NH_2(Cr), (c, d) TpBD-OH, (e, f) MIL@TpBD-ECOEA and (g, h) MIL@MITpBD.

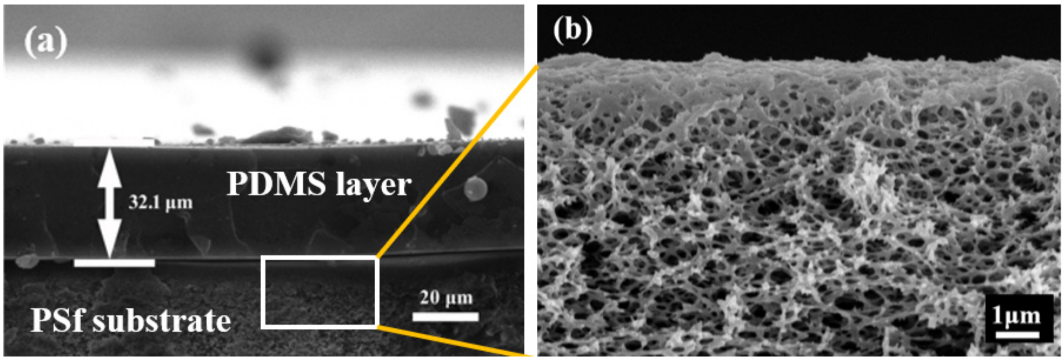

Fig. S5 Cross-sectional SEM images of the PSf substrate near its surface. (a) Low-magnification cross-sectional view of the PSf substrate, reproduced from Figure 4(a) in the main manuscript; (b) High-magnification cross-sectional view of the PSf substrate within the white-boxed region.

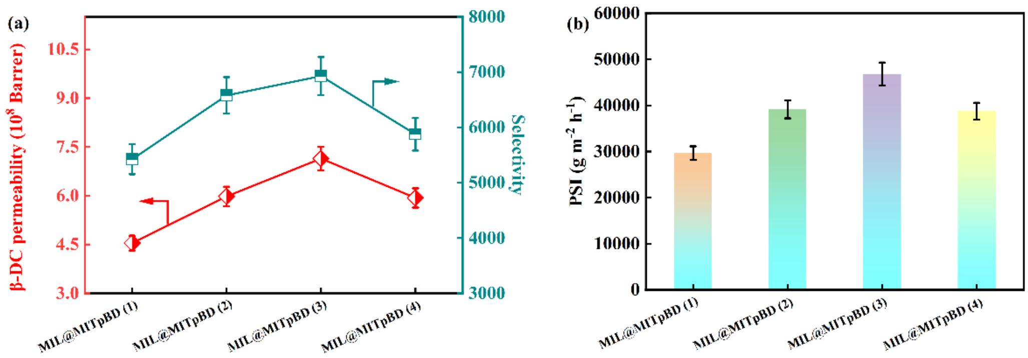

Fig. S6 Impact of the thickness of the imprinted layer on (a) $P_{\beta\text{-DC}}$ and $\alpha_{\beta\text{-DC/H2O}}$, (b) *PSI*.

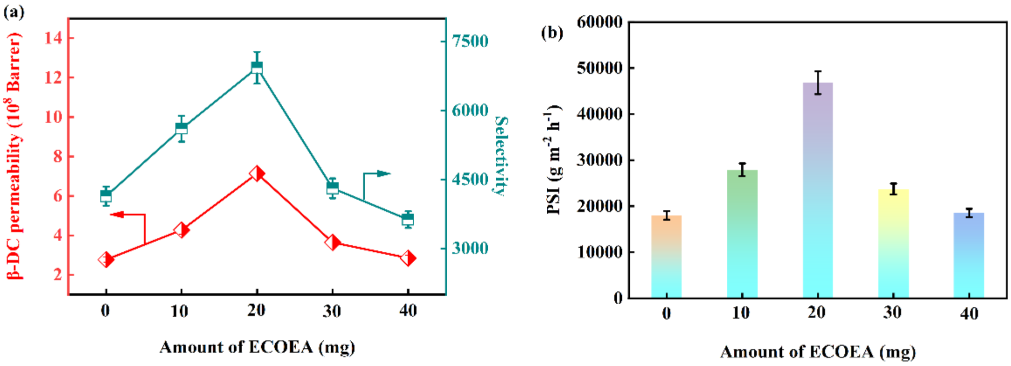

Fig. S7 Impact of ECOEA dosage on (a) $P_{\beta\text{-DC}}$ and $\alpha_{\beta\text{-DC/H2O}}$, (b) *PSI*.

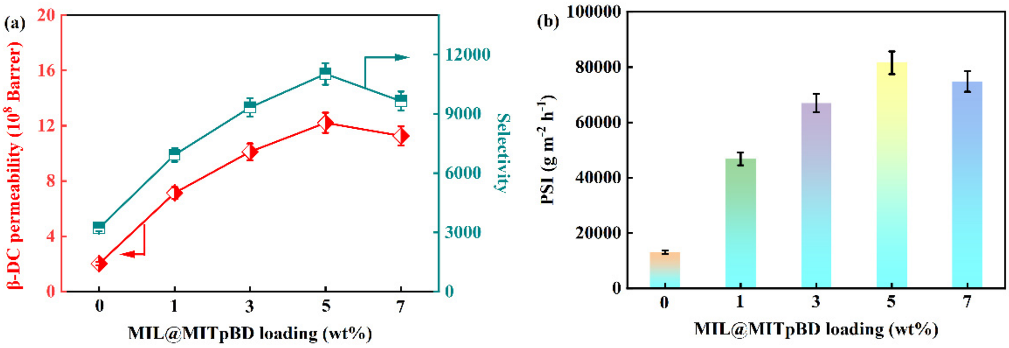

Fig. S8 Impact of MIL@MITpBD loading on (a) $P_{\beta\text{-DC}}$ and $\alpha_{\beta\text{-DC/H2O}}$, (b) *PSI*.

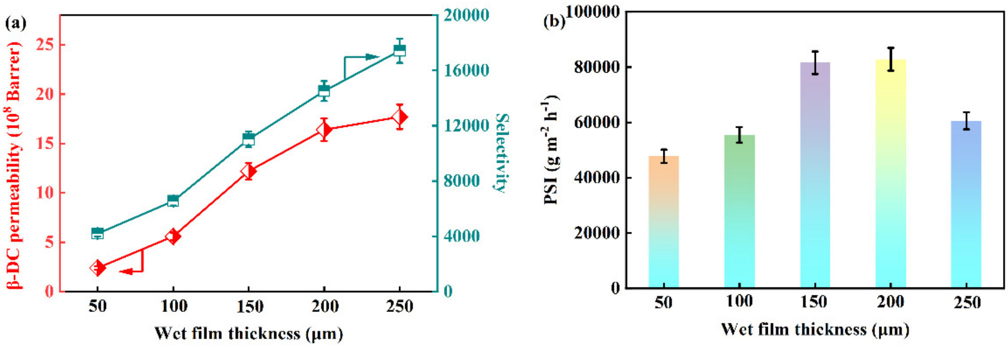

Fig. S9 Impact of wet film thickness on (a) $P_{\beta\text{-DC}}$ and $\alpha_{\beta\text{-DC/H2O}}$, (b) *PSI*.

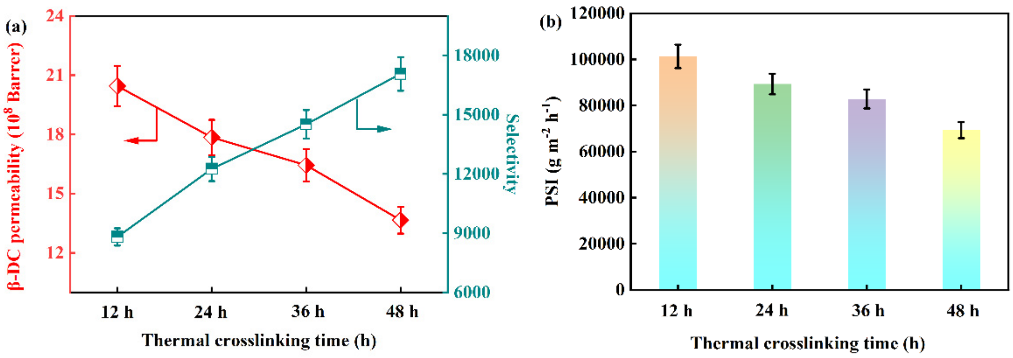

Fig. S10 Impact of crosslinking time on (a) $P_{\beta\text{-DC}}$ and $\alpha_{\beta\text{-DC/H2O}}$, (b) *PSI*.

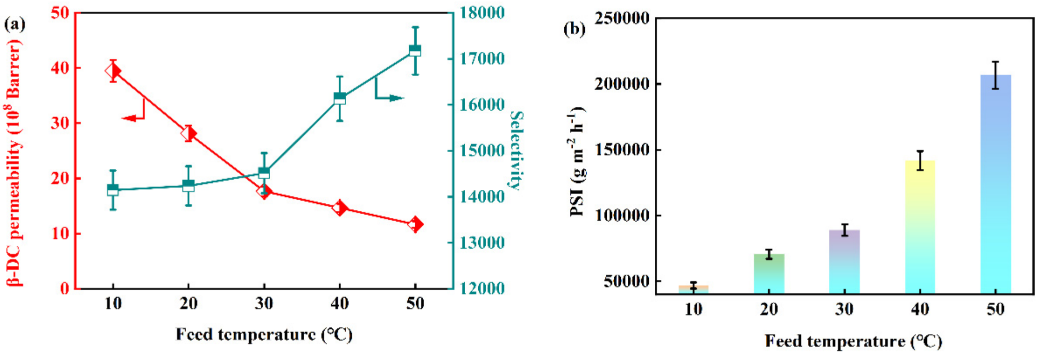

Fig. S11 Impact of feed temperature on (a) $P_{\beta\text{-DC}}$ and $\alpha_{\beta\text{-DC/H}_2\text{O}}$, (b) *PSI*.

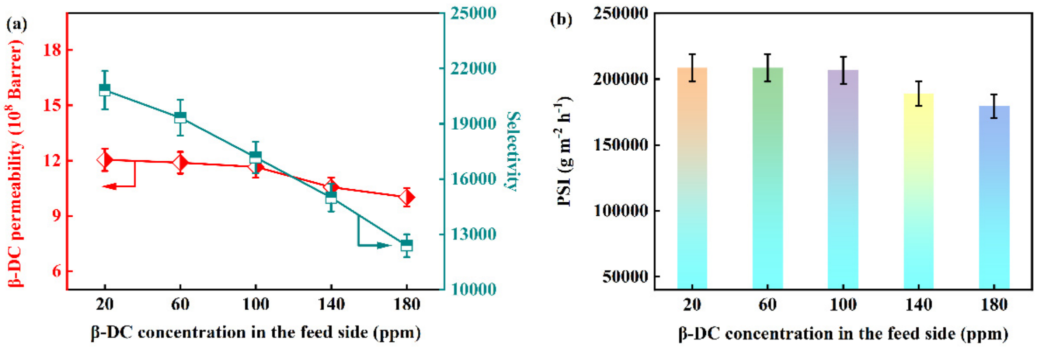

Fig. S12 Impact of β-DC concentration on (a) $P_{\beta\text{-DC}}$ and $\alpha_{\beta\text{-DC/H2O}}$, (b) *PSI*.

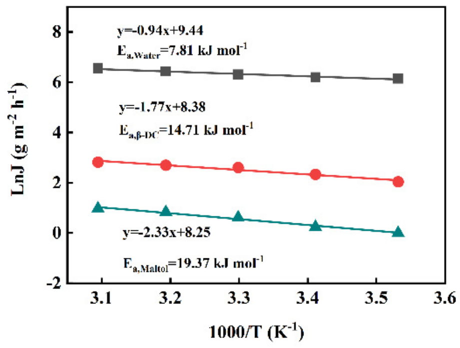

Fig. S13 In ternary system, Arrhenius plots of $J_{\beta\text{-DC}}$, J_{Maltol} and J_{H2O} in PDMS/MIL@MITpBD vs. 1000/T.

Table S1 The pore size distribution parameters of MIL-101-NH_2, TpBD-OH, MIL@TpBD-ECOEA, MIL@NITpBD and MIL@MITpBD.

Sample	BET surface area (m^2 g^{-1})	Total pore volume (cm^3 g^{-1})	Average pore diameter (nm)
MIL-101-NH_2	674.41	0.64	3.81
TpBD-OH	189.65	0.49	10.52
MIL@MITpBD	405.88	1.06	10.42
MIL@TpBD-ECOEA	386.79	1.10	11.39
MIL@NITpBD	380.82	0.89	9.36

Table S2 The characteristic parameters of MIL-101-NH_2, TpBD-OH, MIL@NITpBD and MIL@MITpBD.

Sample	First decomposition Step (℃)	Second decomposition Step (℃)	Third decomposition Step (℃)	T_{d10} (℃)	T_{onset} (℃)	$T_{max}{}^{a}$ (℃)	$T_{max}{}^{b}$ (℃)
MIL-101-NH_2	30-160	178-286	288-317	219	184	191	293
TpBD-OH	30-237	254-383	392-558	293	312	162	507
MIL@NITpBD	30-244	260-330	333-359	276	278	184	338
MIL@MITpBD	30-233	250-332	333-392	278	279	185	336

Table S3 The characteristic parameters of PDMS and PDMS/MIL@MITpBD.

Sample	First decomposition Step (℃)	Second decomposition Step (℃)	T_{d10} (℃)	T_{onset} (℃)	T_{max}^{a} (℃)	T_{max}^{b} (℃)
PDMS	281-390	493-607	344	325	353	561
PDMS/MIL@MITpBD-5	355-689	-	486	517	587	-
PDMS/MIL@MITpBD-7	356-695	-	487	522	597	-

References

19. Lu, Q.; Duan, H.Y.; Zhang, K.; Wang, H.; Han, D.L.; Wang, Z.L.; Xu, Y.Y.; Yi, Q.Y.; Shen, Y.J.; Yan, T.T.; et al. Regulation of intra-nanopore microenvironment in oxygen-rich Covalent Organic Frameworks for enhanced capacitive deionization. *Adv. Funct. Mater.* **2025**, 35, 2507253, doi:10.1002/adfm.202507253.
29. Wang, J.; Liu, Y.P.; Zhang, C.X.; Wang, Q.L. Ultrahigh proton conductivity of encapsulating Metal-Organic Polyhedra in a Metal-Organic Framework composite material and its nanocomposite membrane. *Chem. Mater.* **2025**, 37, 4325-4336, doi:10.1021/acs.chemmater.5c00182.
30. Mao, H.; Zhen, H.G.; Ahmad, A.; Zhang, A.S.; Zhao, Z.P. Fabrication of MOF nanoparticles in PDMS membrane interfacial synthesis for enhanced ethanol permselective pervaporation. *J. Membr. Sci.* **2019**, 573, 344-358, doi:10.1016/j.memsci.2018.12.017.
